# Development and characterization of a standardized adipogenesis assay for testing metabolism disrupting chemicals using human bone marrow derived mesenchymal stem cells

**DOI:** 10.1016/j.namjnl.2025.100029

**Published:** 2025-05-22

**Authors:** Xiao-Min Ren, Richard C Chang, Angélica Amorim Amato, Yikai Huang, Brittanie Yukimtiao, Alexandra Esser, Emma Witteveen, Juliette Legler, Jorke H Kamstra, Bruce Blumberg

**Affiliations:** a Department of Developmental and Cell Biology, University of California, Irvine, CA 92697-2300, USA; b Faculty of Environmental Science and Engineering, Kunming University of Science and Technology, Kunming 650500, China; c Division of Toxicology, Institute for Risk Assessment Sciences, Faculty of Veterinary Medicine, Utrecht University, Utrecht, The Netherlands; d Department of Biomedical Engineering, University of California, Irvine, CA 92697-2300, USA

**Keywords:** Obesity, Metabolism disrupting chemicals, Mesenchymal stromal stem cells, Adipogenesis assay, Immortalized hBM-MSC cell line

## Abstract

Obesity is a global health concern linked to various diseases. Recently, chemicals known as "obesogens" have been identified as significant contributors to obesity, highlighting the need for reliable methods to screen chemicals for their obesity-promoting potential. This study aimed to develop a standardized adipogenesis assay using primary human bone marrow-derived multipotent mesenchymal stromal stem cells (hBM-MSCs) to evaluate the adipogenic potential of chemicals. Lipid accumulation in hBM-MSCs was measured after chemical exposure. Assay conditions including cell confluency, fluorescent staining of neutral lipids and fluorometric detection were optimized and standardized. Using the established method, we evaluated the effects of six selected chemicals on the adipogenic responses of hBM-MSCs from different donors, including an in-house generated immortalized hBM-MSC cell line. Not all donor-derived hBM-MSCs were shown to be suitable for the assay, but we observed comparable results among suitable hBM-MSCs and between primary hBM-MSCs and their immortalized derivative cell line. Comparisons with the well-established 3T3-L1 preadipocyte model revealed a strong correlation with the hBM-MSCs assay. We observed that experimental details such as cell density, fluorescent measurement and cell donors can greatly affect outcomes; this stresses the importance of a well described standard operating procedure (SOP). Our developed test method, including a detailed SOP, paves the way for a validated test methodology that is urgently needed and currently lacking for the identification of obesogenic endocrine disruptors.

## Highlights

• Standardized hBM-MSCs assay to screen chemicals promoting adipogenesis.
• Optimized cell density, fluorescence detection, and assay reproducibility.
• Evaluated six chemicals’ effects on adipogenesis with high assay reliability.
• Established an immortalized hBM-MSC line with results matching primary cells.
• hBM-MSCs assay showed strong correlation with the 3T3-L1 adipogenesis model.

## Graphical abstract

**Figure:**
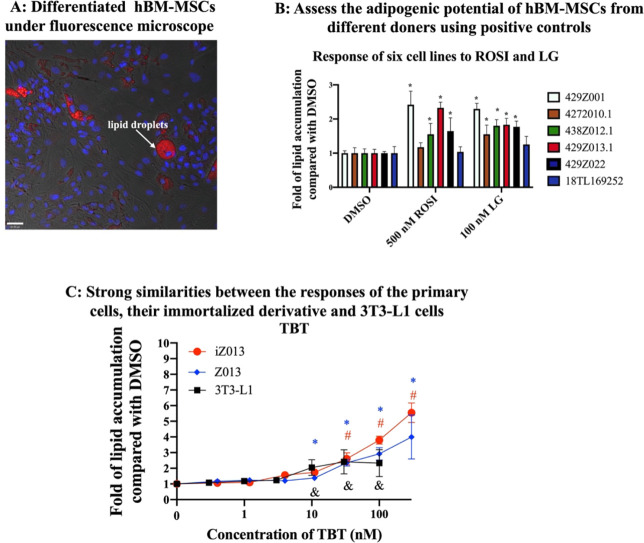

## 1 Introduction

Obesity is closely associated with numerous health problems, including cardio-vascular disease, type 2 diabetes, cancer, osteoporosis, and has become a serious global health issue (Piché et al., 2020; Kahn et al., 2006). According to the World Health Organization, 13 % of adults worldwide are obese, and 43 % are overweight (World Health Organization, 2024). This problem is particularly severe among children and adolescents; the obesity rate for individuals aged 5–19 has increased ten-fold from 1975 to 2022 (World Obesity Federation, 2024). While the causes of obesity are complex and multifaceted, encompassing lifestyle, dietary habits, and genetic factors, exposure to certain chemicals called “obesogens” has recently been shown as an important potential factor for obesity (Heindel et al., 2022). Such obesogens can be natural chemicals, pharmaceutical drugs, and xenobiotic chemicals that promote obesity (Heindel et al., 2024). Obesogens are a subset of a larger group of chemicals called metabolism disrupting chemicals (MDCs) that can affect various aspects of metabolism (Heindel et al., 2017).

To uncover links between obesogens and obesity and reveal the potential mechanisms, it is crucial to develop reliable methods to assess and screen candidate chemicals for their potential to affect obesity-related pathways. There have been a variety of in vivo and in vitro methods developed to study the impact of obesogens on obesity development (Kassotis et al., 2022; Sargis et al., 2010), yet none of those methods are used by chemical regulation. In vivo methods primarily involve exposing animal models to chemicals over a long period and then assessing their effects on entire organism. For example, by exposing mice or rats with chemicals, scientists have observed potential impacts on weight gain, fat accumulation, and the development of metabolic diseases (Panchal and Brown, 2011). In vivo models have many advantages. For example, in vivo studies often provide insights into physiological relevance, systemic responses, and long-term effects that cannot be captured by in vitro methods. Besides, in vivo models also have the advantage to study the interplay between different organ systems in both developing and adult organisms. In vitro methods use cell culture models to examine the effects of chemicals on cells (Poulos et al., 2010). The advantages of in vitro methods include shorter experimental times, lower costs, ease of execution, and allowing for a more detailed understanding of the mechanisms through which obesogens induce obesity.

Obesogens can induce obesity through various mechanisms (Griffin et al., 2020). Prime among these mechanisms are direct effects on adipocytes. These effects include commitment of MSCs to the adipocyte lineage, differentiation of preadipocytes into mature adipocytes, proliferation of adipocytes, modulation of adipocyte lifespan and renewal, lipogenesis/lipolysis as well as thermogenesis in brown and beige adipocytes (Heindel et al., 2017). White adipocytes are essential because they store excess energy in the form of lipids, contributing to body fat accumulation. Researchers typically expose the cell culture models to candidate obesogens and then assess their effects on the processes of adipogenesis. Adipogenesis can be assessed and quantified by measuring endpoints such as the degree of intracellular lipid accumulation, and the expression of adipogenic genes and their corresponding protein products. Currently, the in vitro methods for this aspect mainly employ preadipocyte cell lines (e.g., murine 3T3-L1 cells), uncommitted MSCs and several MSC-like cell lines (e.g., C3H10t1/2 cells) (Kassotis et al., 2022). These cell models can recapitulate the processes of adipogenic differentiation, proliferation, and adipocyte function (e.g., lipolysis) under controlled experimental conditions, which can be used to test the potential contribution of obesogens to the development of obesity and provide insight into underlying mechanisms.

Methodologies that rely on murine models may not fully capture the mechanisms of human adipogenesis, due to species-specific genetic, metabolic, and hormonal differences, as well as variations in fat distribution and immune responses. These differences can lead to discrepancies in how adipogenesis processes are regulated and function in humans compared to mice. For instance, the murine 3T3-L1 adipogenesis assay has shown robustness (Biserni et al., 2019), yet only limited correlations were observed between chemicals and mixtures tested in 3T3-L1 cells vs. human MSCs (Bérubé et al., 2023). These results indicated that human cell models might be more relevant to measure obesogenic action of chemicals. However, within-model discrepancies have also been reported. The ubiquitous environmental pollutant, bisphenol A (BPA), was found to promote adipogenesis of human MSCs at environmentally relevant concentration (1–1000 nmol/L) in one study (Cohen et al., 2021), but showed no significant effect in another (Chamorro-García et al., 2012). These discrepancies likely arose from variations in cell sources, experimental conditions, or methodologies used. Therefore, by ensuring robustness and reproducibility, standardizing human-relevant methodologies will be essential for leveraging human MSCs to explore how chemicals influence adipogenesis and their potential implications for obesity-related health problems.

In this context, the aim of this study was to develop a robust and well-characterized in vitro testing method using human MSCs to screen the capacity of MDCs to induce white adipocyte differentiation. This assay could then be implemented in a larger integrated testing strategy as part of the EU funded Horizon 2020 project GOLIATH, which focusses on the urgent need for testing methods for MDCs, as there are currently no validated testing guidelines for MDCs (Legler et al., 2020).

Human MSCs can be classified into several different types based on their source; these include bone marrow-derived MSCs, adipose-derived MSCs (AD-MSCs), umbilical cord-derived MSCs (UC-MSCs), dental pulp-derived MSCs (DP-MSCs), placenta-derived MSCs (PL-MSCs), amniotic membrane-derived MSCs (AM-MSCs), and synovial membrane-derived MSCs (SM-MSCs) (Andrzejewska et al., 2019). Each type exhibits unique biological properties, differentiation capabilities, and immunomodulatory functions, making them suitable for various research and clinical applications (Pittenger et al., 2019). Human bone marrow sourced MSCs have multiple possible fates, including adipose, bone, and cartilage (Chen et al., 2016). They can serve as indicators of chemicals that promote one fate vs. another.

Bone marrow-derived MSCs can differentiate into adipocytes through a complex and multi-step process involving a network of transcription factors that regulate the expression of genes that promote the adipocyte phenotype (Cristancho and Lazar, 2011; Palou et al., 2000). These include peroxisome proliferator-activated receptor gamma (PPARγ), CCAAT/enhancer-binding proteins (C/EBPs), sterol regulatory element-binding protein (SREBP), and glucocorticoid receptor (GR). Adipocyte differentiation can be induced in cell culture by stimulation of MSCs with isobutylmethylxanthine (IBMX), dexamethasone, and insulin. IBMX inhibits phosphodiesterases, leading to increased intracellular levels of cyclic AMP and activation of C/EBP-δ. Dexamethasone induces expression of C/EBP-β. C/EBP-β and -δ, in turn, these induce the expression of C/EBP-α and PPARγ. Insulin stimulates both adipogenesis and lipogenesis through induction of SREBP-1c and other transcription factors, in addition to directly inhibiting lipolysis. Adipogenesis can be assessed and quantified by measuring endpoints such as the degree of intracellular lipid accumulation, and the expression of adipogenic genes and their corresponding protein products.

Here, we developed an adipogenesis assay involving staining fixed cells with Nile Red for lipid detection and Hoechst 33,342 for DNA content detection, with fluorescence quantified using a microplate reader. We established and verified the adipogenesis assay based on primary hBM-MSCs by using two positive control ligands, the PPARγ agonistic ligand rosiglitazone (ROSI) and the RXR agonistic ligand LGD100268 (LG). We examined cell confluency and detection methods to optimize the assay conditions. Then, we tested the suitability of six primary hBM-MSCs from different donors for the assay and qualified three for use in the assay. Using the established method, we evaluated the effects of six selected chemicals on adipogenesis using cells we qualified for the assay as well as the replicability, repeatability and variability of the assay. To adapt this assay for future high-throughput screening and long-term studies, we immortalized one qualified hBM-MSC, then compared the differences in the response of chemicals between primary cell and the immortalized derivative using RNA sequencing (RNA-seq) and adipogenesis assays. We also compared results of the hBM-MSCs adipogenesis assays with those of the same chemicals in the 3T3-L1 preadipocyte cell adipogenesis assay.

## 2 Materials and methods

### 2.1 Chemicals and reagents

ROSI was from Cayman Chemical Company (USA). LG, dexamethasone, insulin, IBMX, Nile Red, tributyltin chloride (TBT), perfluorooctanoic acid (PFOA), BPA, triphenyl phosphate (TPP), p,p’-dichlorodiphenyldichloroethylene (p,p’-DDE), triclosan (TCS) were purchased from Sigma-Aldrich (USA). Fetal Bovine Serum (FBS) were from Gemini Bio-Products (USA) and Gibco (USA). Formaldehyde, Hoechst 33,342, dimethyl sulfoxide (DMSO), and Minimal Essential Medium α were from Thermo Fisher Scientific (USA). SupplementMix MSC Growth Medium 2 was from PromoCell (Germany). The detail information for the chemicals and reagents are provided in the Supplemental Material.

### 2 HBM-MSCs culture

2

Pure and sterile primary human mesenchymal stem cells sourced from bone marrow were used for the assay. Six cell lines were purchased at passage two from PromoCell (Germany) and Lonza (Switzerland) in cryopreservation solution. The detailed lot information for the cells is as follows: 429Z001-Female, 438Z012.1-Male, 429Z013.1-Male, 4272,010.1-Female, 4292Z022-Male (all Promocell, Germany) and 18TL169252-Female (Lonza, Switzerland).

Upon arrival, cryopreserved hBM-MSCs were immediately stored in liquid nitrogen or seeded at a density of 4000 cells/cm². For seeding, use MSC growth medium (SupplementMix, MSC Growth Medium 1, PromoCell) supplemented with 100 IU/mL penicillin and 100 µg/mL streptomycin. Cells were split or used for seeding at 80–90 % confluency. Once cells were expanded and frozen at passage four, they can be thawed, further expanded, and used in the adipogenesis assay at passage six.

### 2 3 HBM-MSCs adipogenesis assay

A detailed standard operating procedure (SOP) of the hBM-MSCs adipogenesis assay is provided in Supplemental Material (Texts-Part 1). Briefly, hBM-MSCs were cultured in alpha Modification of Eagle’s minimal essential medium (αMEM) (Thermo Fisher Scientific, USA) supplemented with 15 % FBS (Gemini Bio-Products), 2 % penicillin, 5000 IU/mL streptomycin (Corning, USA), and 10 µM HEPES (Fisher Chemical, USA). Cells were split at 90 % confluency and used at passage six for adipogenesis assays. Cells were seeded in the 24-well plate at 40,000 cells per well. When cells were just short of complete confluency, the medium was replaced with differentiation media [αMEM, 15 % FBS, supplemented with adipogenic induction cocktail (MDI: 500 µM IBMX, 1 µM dexamethasone, 5 µg/mL human recombinant insulin)] and ligands. Differentiation medium containing control chemicals or candidate obesogens (dissolved in DMSO) were administered twice weekly for two weeks. A recommended time schedule of this assay is provided in Supplemental Material (Figure S1). DMSO concentration in the medium was kept at 0.1 % and identical between vehicle control and test chemicals. At the end of each assay, cells in the 24-well plates were fixed in 3.7 % formaldehyde in phosphate buffered saline (PBS) buffer for 30 min at room temperature (25 °C) for lipid staining. Fixing the cells with formaldehyde ensures that the intracellular lipids remain stable and are not affected by subsequent experimental procedures.

### 2.4 Lipid accumulation assessment

Cells were ready for intracellular lipid accumulation analysis 14 days after adipo-genic differentiation induction. Lipids were stained with Nile Red, and nuclei were stained with Hoechst 33,342 to normalize lipid staining to cell count. Using Nile Red to stain cells and detect fluorescence is a widely used method for measuring intracellular lipid content. Before staining, cells were fixed in 3.7 % formaldehyde for 30 min at room temperature (25 °C). If phenol red-containing media were used, cells were left in PBS at 4 °C overnight to prevent interference, as phenol red and Nile Red have similar excitation and emission spectra. This overnight step allowed phenol red to be released from the cells.

After fixation, the fluorescence background was measured. The excitation/emission wavelengths for Hoechst 33,342 and Nile Red were 355/460 nm and 485/590 nm, respectively. Cells were stained with 5 µg/mL Hoechst 33,342 and 1 µg/mL Nile Red for 30 min at room temperature. Then, the staining solution was removed, and cells were washed twice with PBS. When optimizing the detection point number of this assay, the fluorescence of both Nile Red and Hoechst was measured by detecting 1, 9, 25 and 400 points per well using a fluorescence plate reader (Tecan, USA) using a bottom read well scan. For other parts of this study, the fluorescence was measured by detecting 400 points per well. Intracellular lipid accumulation was calculated by normalizing Nile Red relative fluorescence units (RFU) to Hoechst 33,342 RFU. For each well, the average background fluorescence was subtracted from the corresponding Nile Red and Hoechst fluorescence. Finally, the corrected Nile Red fluorescence was divided by the corrected Hoechst fluorescence to determine lipid accumulation.

### 2.5 Immortalization of HBM-MSCs

We immortalized a hBM-MSC cell line (429Z013.1-Male) as follows. hBM-MSCs were seeded in 6-well plates at a density of 6 × 104 cells/well and grown in growth medium (MEM alpha with 15 % FBS) for 12 h to reach ∼30 % confluence. Then, 1 mL of infection medium containing 1 × 106 copies of Lenti-hTERT virus (abm, Canada) was used for immortalization. After 24 h, cells were maintained in growth medium for another 48 h to complete infection. For drug selection, 1 % puromycin in growth medium was used for 7 days. Six to twelve single cells were randomly picked and grown individually in 96-well plates for 7 to 10 days, then transferred to 6-well plates using trypsin. After 7 days, once cells reached confluence in 6-well plates, T75 flasks were used to expand cell stocks.

To characterize and determine the multipotency of immortalized hBM-MSCs, cell surface markers of mesenchymal stem cells were employed. According to the standards proposed by the Mesenchymal and Tissue Stem Cell Committee of the ISCT for defining hBM-MSCs in laboratory-based scientific and preclinical studies, we used the MSC Phenotyping kit (Miltenyi Biotec, Germany) to test for presence of CD73, CD90, and CD105. RNA sequencing (RNA-seq) was employed to distinguish transcriptomal differences between original hBM-MSCs vs. immortalized hBM-MSCs after 14 days adipogenesis. Additionally, the adipogenic potential of immortalized hBM-MSCs was determined by a 14-day adipogenesis assay.

### 2.6 RNA-seq analysis of primary vs. immortalized 429Z013.1-Male cells

Primary hBM-MSCs and immortalized hBM-MSCs were seeded in six-well plate and the standard 14-day adipogenesis assay performed with differentiation media [αMEM, 15 % FBS, supplemented with adipogenic induction cocktail plus positive control ligand (500 nM ROSI). Total RNA samples were extracted using Direct-zol RNA Miniprep Kits (Zymo Research). RNA sequencing was performed by UCI Genomics High-Throughput Facility. Pariedend reads were subjected to quality control using FastQC to assess the integrity and quality of the RNA. Following quality control, adapter sequences and low-quality bases were trimmed using Trimmomatic to ensure high-quality reads for downstream analysis. The trimmed reads were then aligned to the reference genome using the STAR Aligner. The alignment process was optimized to ensure high accuracy and sensitivity, allowing for the identification of both canonical and non-canonical splice junctions. Post-alignment, transcript quantification was performed using RSEM and FastQx. These tools provided accurate quantification of transcript abundance by accounting for the uncertainty in read assignments. Differential expression analysis was conducted using DESeq2. This statistical tool was employed to identify differentially expressed genes (DEGs) between the experimental conditions. DESeq2 accounts for variability within the data and adjusts for multiple testing, providing a robust set of DEGs for further analysis.

### 2.7 Adipogenesis assay of 3T3-L1 cells

Detailed information for the standard 3T3-L1 adipogenesis assay can be found in Supplemental Material (Texts – Part 2). Briefly, 3T3-L1 preadipocytes (ATCC, Manassas, VA) were maintained in pre-adipocyte media [DMEM with high glucose, GlutaMAX, and phenol red (Gibco), supplemented with penicillin and streptomycin (10,000 U/mL; Gibco) and 10 % bovine calf serum (ATCC)]. When cells reached 70–80 % confluency, they were subcultured using 0.05 % trypsin-EDTA (Gibco). All experiments were performed at passage six, as 3T3-L1 cells tend to lose differentiation capacity at higher passages. Cells were seeded in pre-adipocyte media and grown to confluency, then allowed to undergo 48 h of growth arrest. Differentiation was initiated using test chemicals in differentiation media [DMEM with 10 % FBS (Gibco), 1 % penicillin/streptomycin, 1.0 μg/mL human insulin, and 500 μM IBMX]. After 48 h, the medium was replaced with fresh dilutions of test chemicals in adipocyte maintenance media (differentiation media without IBMX), refreshed every 2–3 days until the end of the assay. At terminal differentiation (day 8), cells were fixed in 3.7 % formaldehyde in PBS and stained with 1 μg/mL Nile Red (Sigma) and 1 μg/mL DAPI (Thermo Fisher) in 250 μL staining buffer. Adipogenesis was quantified using a Tecan fluorimeter to measure Nile Red and DAPI fluorescence by bottom-well scanning.

### 2.8 Gene expression analysis of 3T3-L1 cells and hBM-MSCs

For gene expression analysis of 3T3-L1 cells and hBM-MSCs, total RNA was isolated using the Nucleospin® RNA isolation kit (Machery-Nagel, Netherlands). cDNA was synthetized using the High-Capacity cDNA Reverse Transcription Kit (Thermo Fisher). For the hBM-MSCs, a primer set for PPAR related genes was taken from another study in which those primers were already validated (Chamorro-García et al., 2012). Primers for all other human genes were designed using the Primer-BLAST online tool by the National Center for Biotechnology Information (NCBI). For the 3T3-L1 cells, primers sets were taken from another study in which those primers were already validated (Kamstra et al., 2014). The details of the sequences of primers used for quantitative real-time reverse transcriptase polymerase chain reaction (qPCR) are described in Supplemental Material (Table S1 and Table S2). qPCRs were performed on BioRad thermal cyclers (CFX96, BioRad, Netherlands). Each treatment was studied in triplicate. Two independent experiments were performed per treatment. Results were normalized by two reference genes NONO and beta ACTIN, which showed stability over all samples.

### 2.9 Statistical analysis

The data are presented as mean ± standard error of the mean (*n* = 3). Significance of the effects induced by the chemicals compared to the control group in hBM-MSCs adipo-genesis assay was determined using one-way analysis of variance (ANOVA) followed by a least significant difference multiple comparisons test. Significance of the effects induced by the ROSI or LG compared to the DMSO group in hBM-MSCs adipo-genesis assay was determined using T-Test (Unpaired, Two-tailed). Significance of the effects induced by the DMSO or 500 nM ROSI in different cell density was determined using ANOVA followed by a least significant difference multiple comparisons test. Statistical significance was defined as *p* < 0.05. GraphPad Prism 9.0 (GraphPad Software Inc., USA) was used for all statistical analyses.

To investigate the replicability and repeatability of the assay, we tested the adipogenic effects of ROSI (a positive control ligand) and the six selected chemicals using one cell line (429Z001-Female) across three separate experiments (biological replicates, all experiments were repeated three times). Using the results of ROSI and six selected chemicals from three replicate wells (technical replicates, results were obtained from one experiment), we determined the experimental replicability of our assay. Using the results of the effects at highest concentrations tested in three separate experiments, we determined the experimental replicability of our assay.

## 3 Results

### 3.1 Morphology of hBM-MSCs before and after adipogenesis

**Fig. 1:**
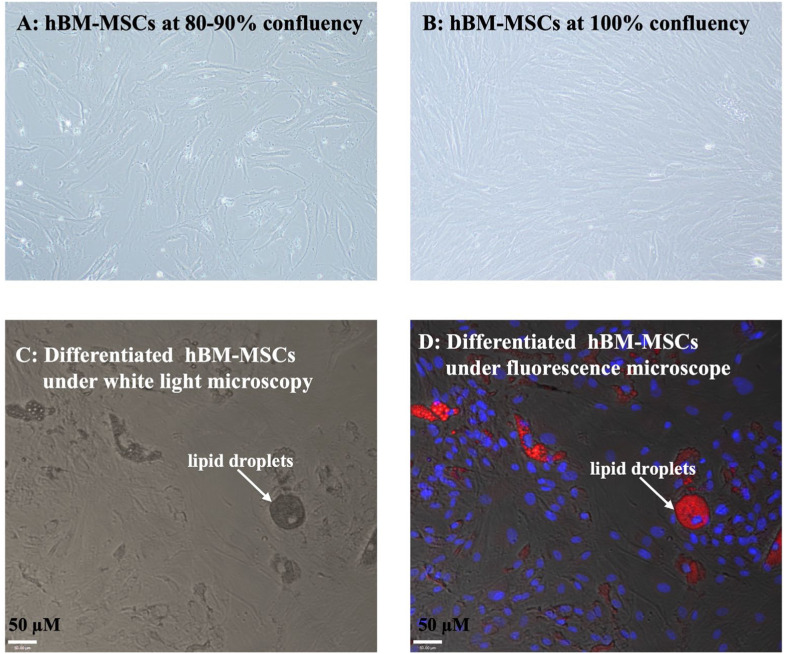
Morphological changes in hBM-MSCs before and after adipogenic differentiation, illustrating cell confluency and lipid accumulation. A: hBM-MSCs (429Z001) at 80–90 % confluency; B: hBM-MSCs at full (100 %) confluency; C: Morphology of differentiated hBM-MSCs under bright-field microscopy; D: Fluorescence microscopy image of differentiated hBM-MSCs stained with Nile Red and Hoechst 33,342, where blue indicates cell nuclei (Hoechst) and red indicates lipid droplets (Nile Red). All images were captured at 20× magnification.

Morphological assessment is essential for the initial identification and ongoing monitoring of hBM-MSCs cultures. hBM-MSCs typically exhibit a fibroblast-like, spindle-shaped morphology, characterized by elongated bodies with tapered ends. Fig. 1A shows the morphology of hBM-MSCs (429Z001-Female) at 80–90 % confluency (under 20× magnification). After seeding, cells were allowed to grow to 100 % confluency over 2–3 days. Fig. 1B shows the morphology of hBM-MSCs after reaching 100 % confluency. Following treatment with the MDI cocktail and a positive control ligand (500 nM ROSI) for 14 days, some hBM-MSCs differentiated into adipocytes, accumulating lipid droplets and changing from the initial spindle shape to a more rounded form with numerous lipid droplets. Fig. 1C shows the morphology under bright field illumination, with dark areas indicating lipid droplets in differentiated adipocytes. Fig. 1D shows the morphology under fluorescence microscope after staining with Nile Red and Hoechst 33,342, where blue represents cell nuclei stained with Hoechst, and red indicates lipid droplets stained with Nile Red.

### 3.2 Impact of cell confluency on the adipogenesis assay

We tested the impact of various factors involved in the adipogenesis assay, which may influence the accuracy of the results. First, we investigated the impact of cell confluency. After seeding the hBM-MSCs (429Z001-Female) in the plate, the cells were allowed to grow to 100 % confluency (2–3 days) before inducing them with the MDI cocktail and ligands in the media. However, determining 100 % confluency is subjective and may vary between assay performers. Therefore, we tested the effect of cell confluency on the accuracy of this assay by seeding different cell densities into a 24-well plate (ranging from 20,000 cells/well to 80,000 cells/well) and using 500 nM ROSI as a positive control ligand to induce adipogenesis. The fold increase in lipid accumulation induced by ROSI over MDI alone was used to investigate the influence of cell confluency on adipogenesis.

**Fig. 2:**
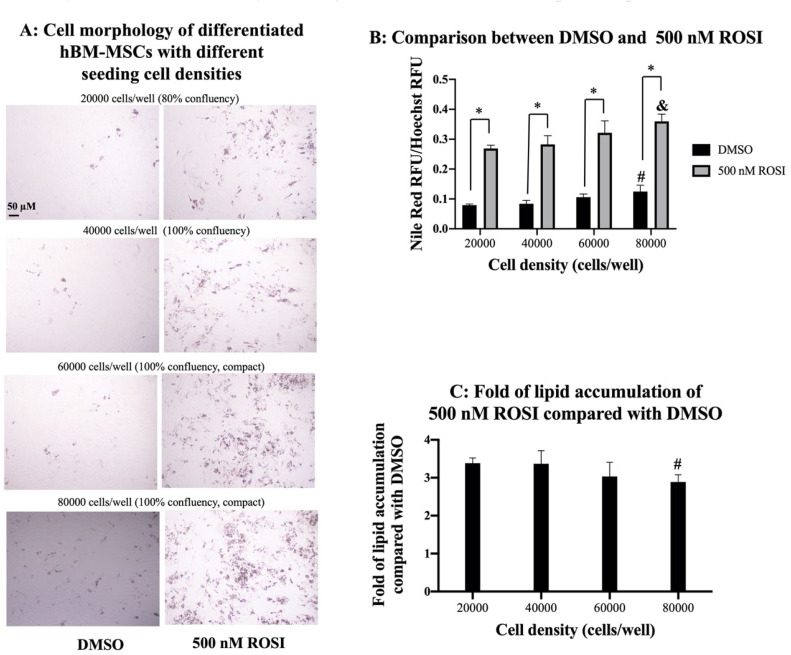
Impact of cell confluency on the hBM-MSC adipogenesis assay, indicating that seeding densities resulting in >100 % confluency did not improve assay performance. A: Representative morphology of differentiated hBM-MSCs at various seeding densities (dark regions indicate lipid droplets in adipocytes); B: Comparison of Nile Red RFU normalized to Hoechst RFU between DMSO and 500 nM ROSI treatment across different seeding densities; C: Fold induction of lipid accumulation by 500 nM ROSI relative to DMSO at each seeding density. * indicates *p* < 0.05 vs*.* DMSO group at the same cell density (unpaired, two-tailed T-test); #indicates *p* < 0.05 vs*.* 20,000 cells/well group within the DMSO condition; & indicates *p* < 0.05 vs*.* 20,000 cells/well group within the 500 nM ROSI condition; Significance between DMSO and ROSI groups was assessed using an unpaired, two-tailed T-Test. Differences among cell densities were analyzed using one-way ANOVA followed by least significant difference multiple comparisons test.

Three days after seeding, the wells with 20,000 cells were about 80 % confluent, while the wells with 40,000 to 80,000 cells/well reached ∼100 % confluency. Compared to the wells with 40,000 cells/well, which were judged as exactly 100 % confluency, the wells with 60,000 or 80,000 cells/well were also judged to be 100 % confluent but appeared more compact. After 14 days of differentiation, all of the 500 nM ROSI-treated groups exhibited significantly more lipid accumulation (the dark areas indicating lipid droplets in differentiated adipocytes) than their corresponding DMSO vehicle groups (Fig. 2A). During the MSC differentiation induction process, the DMSO Control group was treated with MDI cocktail, while the ligand-treated groups received a combination of the MDI cocktail and ligands. The lipid accumulation fold increase of the ligands was determined by comparing the lipid accumulation in the ligand-treated cells with that of the DMSO Control group. For the DMSO Control group, excessive differentiation of cells would result in a high background, making the lipid differentiation fold increase in the ligand-treated group appear lower and affecting the sensitivity of the method. Therefore, it is essential to control the level of adipogenesis in the Control group under appropriate conditions. Background lipid accumulation in the DMSO vehicle groups increased with the initial cell density (Fig. 2B). Similarly, the lipid accumulation in the 500 nM ROSI groups also increased (Fig. 2B). When calculating the fold increase in lipid accumulation in the 500 nM ROSI groups compared to their corresponding DMSO vehicle groups, the fold change decreased by about 20 % (Fig. 2C). Compared to the 40,000 cells/well condition, under the 80,000 cells/well condition, 500 nM ROSI indeed induces more cells with large fat accumulations. However, under the 80,000 cells/well condition, DMSO also induces more cells with fat accumulation, which increases the background signal and consequently reduces the induction fold of 500 nM ROSI. For this reason, we ultimately chose the 40,000 cells/well condition as the optimal condition. Based on these results, we infer that the confluency of cells at the start of differentiation can affect the dynamic range of this assay. More cells in the well or >100 % confluency had no advantage for the assay. Therefore, we recommend seeding 40,000 cells/well in standard 24-well tissue culture plates and inducing hBM-MSCs with the MDI cocktail and ligands when the cells are just short of complete confluency. Our recommendation is derived from a characterized cell line and may require optimization when applied to other cellular models. Given the inherent heterogeneity across cell lines, we strongly recommend empirically determining the optimal seeding density for each new cellular system.

### 3.3 Optimization of detection point number of fluorescence plate reader

Given that hBM-MSCs differentiate into adipocytes with differing levels of lipid accumulation across each well, it is critical to capture a representative signal of the entire cell sample in one well. In our investigation of the detection methods used in the hBM-MSCs adipogenesis assay, we explored the impact of the number of detection points per well on the reliability of the results. Most fluorescence plate readers can detect different numbers of points per well. We compared the results by detecting 1, 9, 25, and 400 points per well with a bottom read scan. We used 500 nM ROSI and 100 nM LG as positive control ligands to induce adipogenesis of hBM-MSCs (429Z001-Female). We found that single-point detection mode exhibited high variation and was unreliable, whereas the 9, 25, and 400-points per well detection modes yielded consistent results with lower variability (Figure S2). Therefore, to ensure reliable data with a reasonable speed, we recommend detecting at least 9 points per well, which balances accuracy, efficiency and speed in the detection process.

### 3.4 Investigation of the stability of staining signals

Another critical aspect of our assay optimization was evaluating the stability of staining signals over time. Typically, we recommend detecting cell samples on the same day they are stained. However, practical reasons may delay detection. We compared the fluorescence signals of cell samples detected immediately after staining with those stored at 4 °C and covered with tin foil for 10 days. 500 nM ROSI and 100 nM LG were used to induce adipogenesis of hBM-MSCs (429Z001-Female). Signals from Nile Red and Hoechst were unchanged after 10 days of storage (Figure S3A-B); fold change in lipid accumulation relative to DMSO vehicle controls also remained unchanged (Figure S3C). These results indicated that with proper storage conditions, the results can be reliably measured up to 10 days post-staining, providing flexibility in experimental workflows and ensuring the robustness of the assay results.

### 3.5 Application of the hBM-MSCs adipogenesis assay to test candidate obesogenic chemicals and investigation of the reproducibility of the assay

**Fig. 3:**
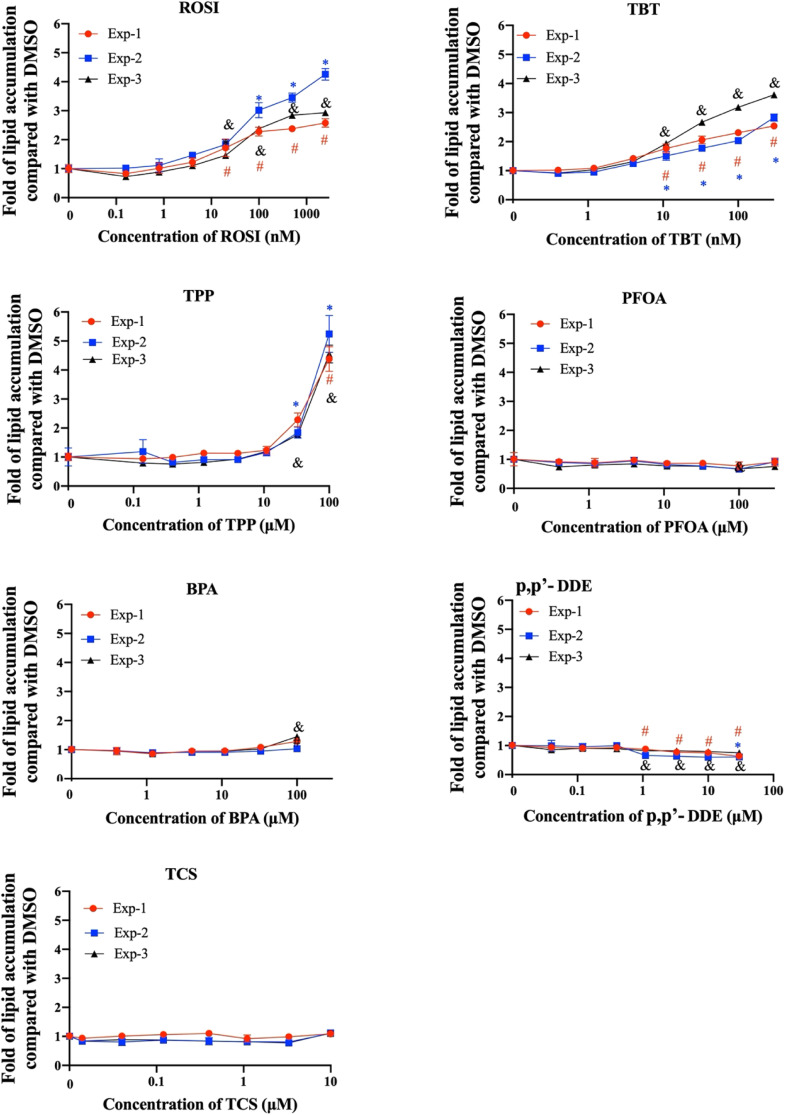
Adipogenesis assay results for ROSI and six selected chemicals using hBM-MSC line 429Z001-Female across three independent experiments, demonstrating good assay replicability and repeatability. Symbols denote statistically significant differences compared to the DMSO vehicle control within each respective experiment: # indicates *p* < 0.05 in Experiment 1; *indicates *p* < 0.05 in Experiment 2; & indicates *p* < 0.05 in Experiment 3. Statistical significance of chemical-induced effects was determined using one-way ANOVA followed by least significant difference multiple comparisons test.

Using the established method, we evaluated the effects of six selected chemicals which were known or suspected MDCs and had been agreed as a core set of chemicals to be tested within the GOLIATH project (Legler et al., 2020) on adipogenesis and investigated the replicability and repeatability of the assay based on these results. We tested the adipogenic effects of ROSI (a positive control ligand) and the six selected chemicals using one cell line (429Z001-Female) across three separate experiments (Fig. 3). Each chemical was tested at seven different concentrations, with ROSI undergoing a five-fold dilution and the other chemicals a three-fold dilution. The highest tested concentrations depended on visual inspection of cytotoxicity or the formation of precipitates. TBT, TCS and BPA were tested up to 300 nM, 10 and 100 µM respectively due to cytotoxicity at higher concentrations, whereas the other three chemicals were tested at their maximum soluble concentration (PFOA (300 µM), TPP (100 µM), p,p’-DDE (30 µM)).

The results indicated that ROSI consistently induced a concentration-dependent increase in intracellular lipid accumulation, demonstrating the sensitivity and efficacy of the assay. Among the six tested chemicals, TBT was particularly notable for its consistent concentration-dependent effect on lipid accumulation across all experiments, with the lowest effective concentration (LOEC) observed at 11 nM. TPP and BPA also elicited significant lipid accumulation responses, with TPP effective at 11 μM and BPA at 100 μM. PFOA, p,p’-DDE, and TCS did not significantly affect lipid accumulation, with p,p’-DDE shows a inhibitory effect on adipogenesis (Fig. 3). Using the results of ROSI and six selected chemicals from three replicate wells, we determined the experimental replicability of our assay, with relative standard deviations (RSD) ranging from 2 % to 27 %. Using the results of the effects at highest concentrations tested in three separate experiments, we determined the experimental replicability of our assay, with RSD ranging from 8 % to 25 %. The above results suggest the good replicability and repeatability of the assay. We defined a successful assay as one where ROSI (500 nM) elicits at least 1.5-fold induction in a statistically significant manner. A positive test chemical is defined as increasing lipid accumulation by at least 1.5-fold in two consecutive dilutions of a factor 3 over the DMSO vehicle control. The 1.5-fold increase in lipid accumulation was selected as a threshold based on our experimental validation, where this level reliably distinguished between positive and negative controls. While smaller effects may be detected with this method, this threshold was chosen to ensure the robustness and reproducibility of the results. The use of two consecutive dilutions provides an additional layer of confidence in the results and helps mitigate any experimental variability. According to this criterion, TBT and TPP are judged as two positive chemicals.

### 3.6 Selection of eligible hBM-MSCs from six different sources

**Fig. 4:**
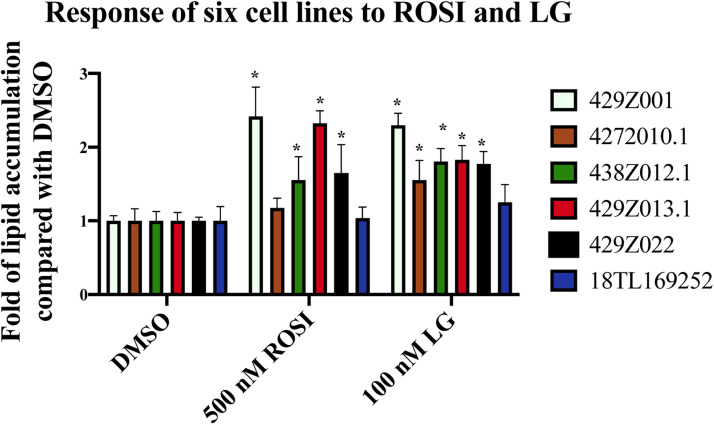
Adipogenic response of hBM-MSCs from six different donors to 500 nM ROSI and 100 nM LG, indicating that three cell lines were suitable for use in this assay. *indicates *p* < 0.05 vs*.* DMSO vehicle control. Statistical significance of the effects induced by ROSI or LG compared to DMSO was determined using an unpaired, two-tailed T-Test.

The adipogenic potential of hBM-MSCs can vary based on their source. Thus, it is essential to test this capability when using new hBM-MSCs from different donors and suppliers. In addition to the 429Z001-Female cells used in the above experiments, we tested five other lots of primary hBM-MSCs from two vendors. To assess the quality and adipogenesis potential of these cells, we tested their responses to two positive control ligands, ROSI and LG. As shown in Fig. 4, 429Z001-Female, 438Z012.1-Male, and 429Z013.1-Male all showed positive responses to these two ligands. Specifically, 429Z001-Female showed fold increases of 2.41 and 2.29 for 500 nM ROSI and 100 nM LG, respectively. 438Z012.1-Male showed fold increases of 1.55 and 1.80, respectively. 429Z013.1-Male showed fold increases of 2.32 and 1.83, respectively. The other three cell lots showed defects in the adipogenesis assay. 4272,010.1-Female and 18TL169252-Female did not respond to ROSI. The 4292Z022-Male cells, while responsive to ROSI, but exhibited different cell morphology from the other batches and had more cellular debris in each well (data not shown). Therefore, we concluded that 429Z001-Female, 438Z012.1-Male, and 429Z013.1-Male cells were eligible for this assay, whereas the others were not deemed suitable for future use due to their non-responsive to ROSI or unnormal cell morphology. The exclusion of non-responsive cell lines could strengthen the reliability of the assay by focusing on the most promising lines.

### 3.7 The variability of the hBM-MSCs adipogenesis assay

**Fig. 5:**
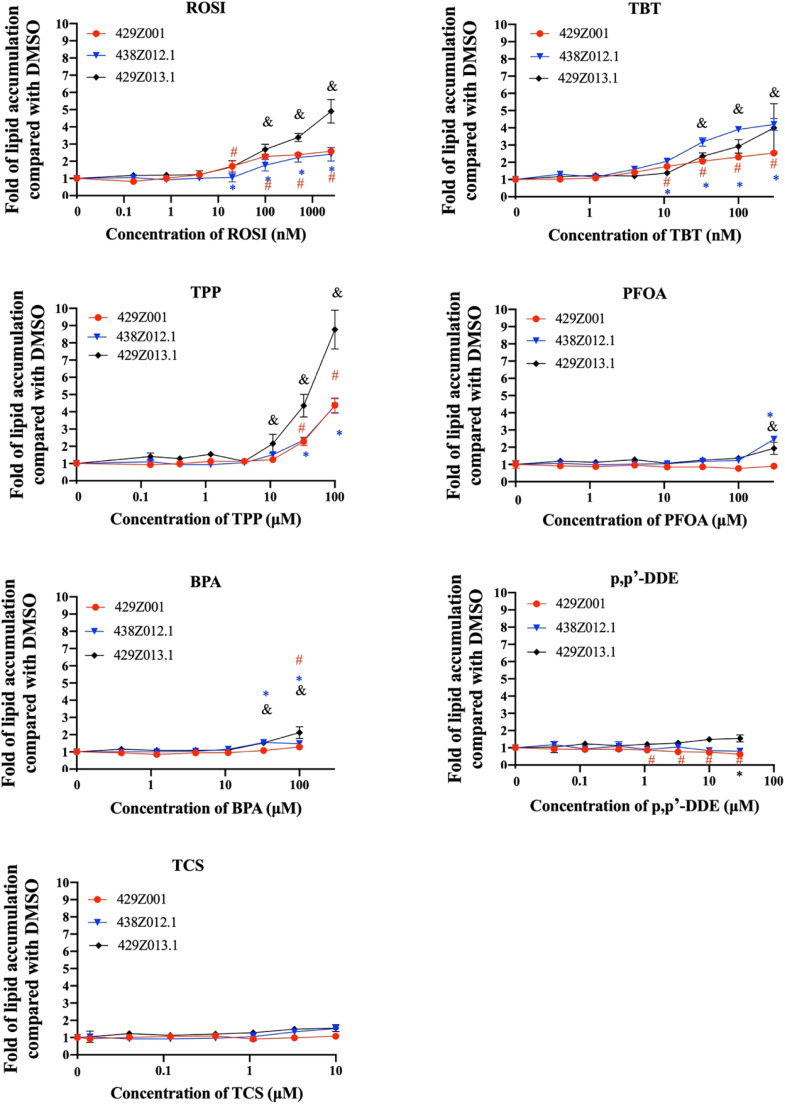
Adipogenesis assay results for ROSI and six selected chemicals using three different hBM-MSC lines, showing generally consistent responses across cell lines. Symbols indicate statistically significant differences compared to the DMSO vehicle control within each respective cell line: # indicates *p* < 0.05 in experiments using 429Z001-Female cells; *indicates *p* < 0.05 in experiments using 438Z012.1-Male cells; & indicates *p* < 0.05 in experiments using 429Z013.1-Male cells. Statistical significance of chemical-induced effects was assessed using one-way ANOVA followed by least significant difference multiple comparisons test.

The hBM-MSCs used in this assay are primary cells obtained from different anonymous human donors, resulting in potential variability in their performance. To assess the variability of the hBM-MSCs adipogenesis assay, the three qualified cell batches noted above (429Z001-Female, 438Z012.1-Male, and 429Z013.1-Male) were used. We compared the effects of ROSI and the six selected chemicals (Legler et al., 2020) by using these three different cell lines (Fig. 5). ROSI consistently increased intracellular lipid accumulation in a concentration-dependent manner across all three cell lines with the LOEC at 20 nM or 100 nM. Similarly, TBT also consistently increased intracellular lipid accumulation in a concentration-dependent manner across all three cell lines with the LOEC at 11 nM. TPP and BPA also showed effective lipid accumulation responses at 11 μM for TPP and 33 μM or 100 μM for BPA. Interestingly, PFOA exhibited varying effects. It had a slight negative impact on the 429Z001-Female cell but a positive effect on the two male cell lines (438Z012.1-Male and 429Z013.1-Male) at a concentration of 300 μM. p,p’-DDE and TCS did not induce significant lipid accumulation in any of the cell lines; although, p,p’-DDE decreased lipid accumulation at four tested concentrations in 429Z001-Female cells (Fig. 5). Overall, the three cell lines yielded consistent results for ROSI, TBT, TPT and TCS but non-consistent results for PFOA, BPA and p,p’-DDE. While we aimed to select the most responsive cell lines for testing, it is important to acknowledge that biological variability exists, and the effectiveness of certain chemicals may be cell-line specific. Further research is needed to understand the underlying mechanisms contributing to this variability.

### 3.8 Comparing the RNA-seq analysis and adipogenesis assay results between primary and immortalized hBM-MSCs

Primary hBM-MSCs cells exhibit significant donor variability, limited lifespan, and inconsistent responses, making immortalized cells more suitable for reproducible and reliable research due to their stable genetic background and extended lifespan. Two cell lines (438Z012.1-Male and 429Z013.1-Male) showed consistent response to the tested chemicals. We chose one of them for immortalization. Here, we immortalized 429Z013.1-Male hBM-MSC cell line to assess whether it could be used in the adipogenesis assay instead of primary cells. We employed RNA-seq analysis to examine the transcriptional similarity and performed adipogenesis assays to compare the adipogenic potential of primary vs. immortalized cells.

**Fig. 6:**
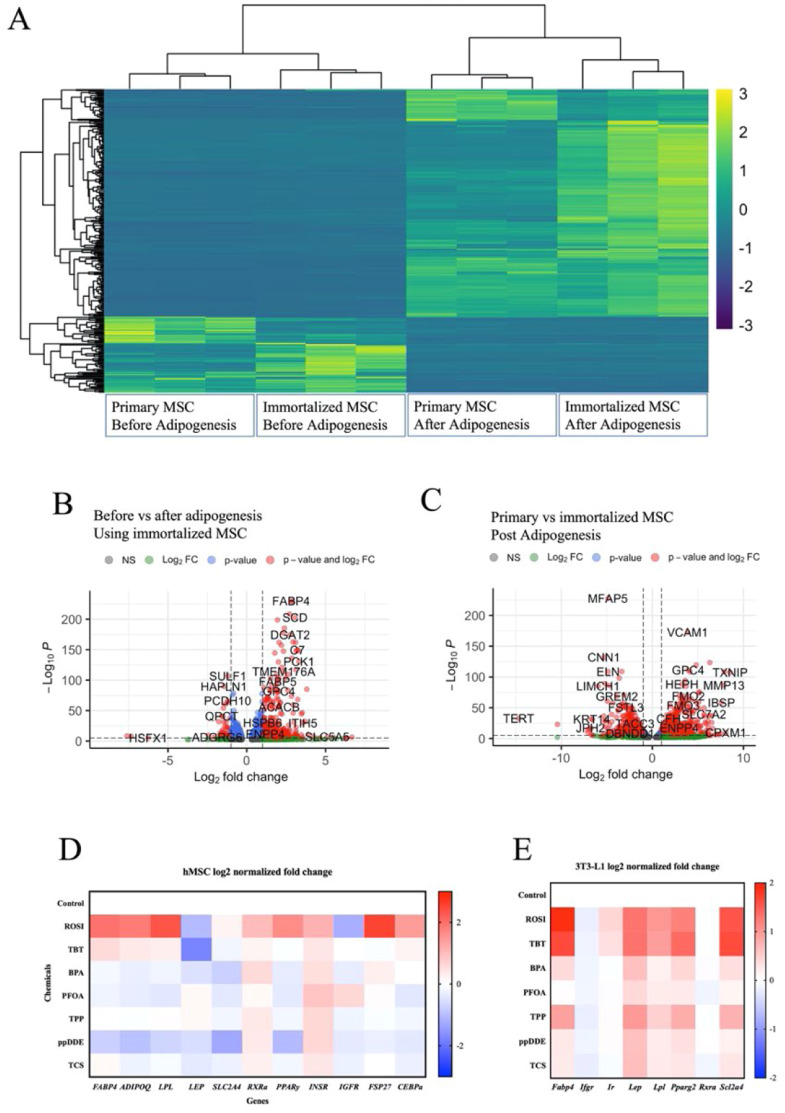
Comparative RNA-seq analysis of primary and immortalized 429Z013.1-Male hBM-MSCs, as well as 3T3-L1 cells, supporting the feasibility of using immortalized cells in the adipogenesis assay. A: Heatmap showing gene expression clusters in primary and immortalized 429Z013.1-Male cells before and after 14 days of adipogenic induction. B: Volcano plot showing differentially expressed genes in immortalized 429Z013.1-Male cells before vs. after 14 days of adipogenic induction. C: Volcano plot comparing gene expression between primary and immortalized 429Z013.1-Male cells after 14 days of adipogenic induction. D: Heatmap of gene expression in primary 429Z013.1-Male cells following chemical exposure. E: Heatmap of gene expression in 3T3-L1 cells following chemical exposure.

Both primary cells and their immortalized derivative showed significant differentially-expressed gene clusters before and after 14-day of adipogenic induction, while there was little difference between the two cell types according to heatmap analysis (Fig. 6A). When visualizing differentially expressed genes using volcano plots, we found adipo-genesis related genes including fatty acid-binding protein 4 (FABP4), stearoyl-CoA de-saturase (SCD), and diacylglycerol O-acyltransferase 2 (DGAT2) were significantly increased after 14-day adipogenesis in the immortalized cells, suggesting a successful adipogenesis induction of this cell line (Fig. 6B). Although some differentially expressed genes existed between primary vs. immortalized cells after 14-day of adipogenesis, these genes were not associated with adipogenic gene clusters (Fig. 6C). Overall, the results of RNA-seq analysis demonstrated transcriptional similarity between primary cells and their immortalized derivative in response to adipogenic induction.

**Fig. 7:**
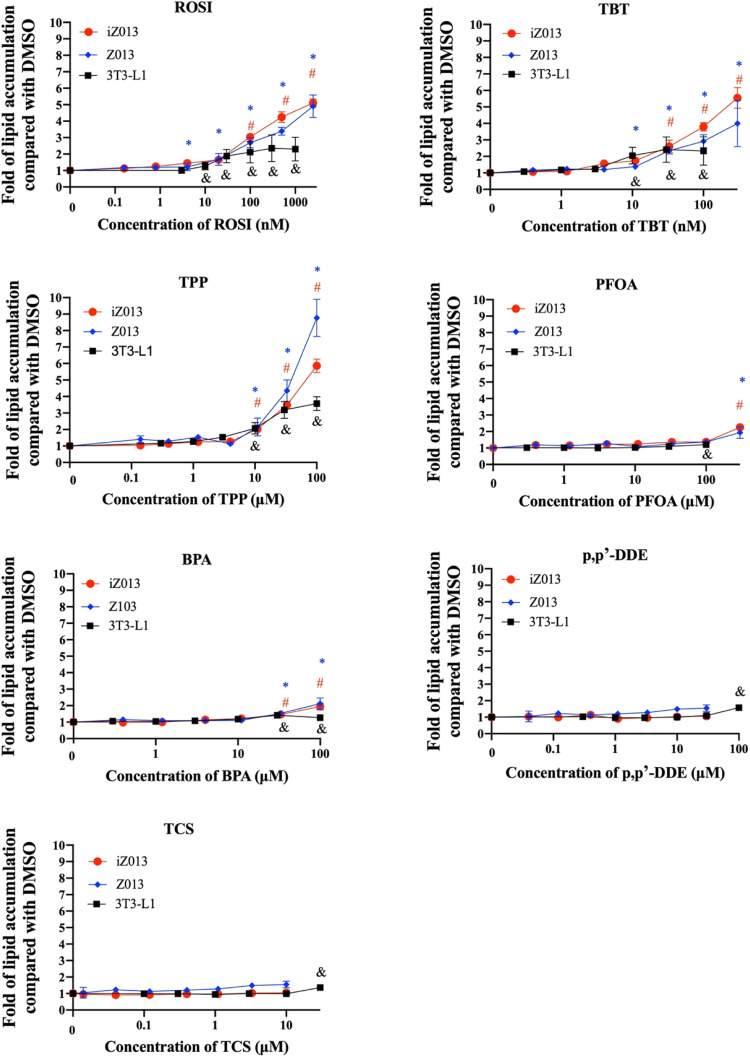
Comparison of adipogenesis assay results across primary 429Z013.1-Male cells (Z013), immortalized 429Z013.1-Male cells (iZ013), and 3T3-L1 cells, supporting the feasibility of using immortalized hBM-MSCs in the assay. Symbols indicate statistically significant differences compared to the DMSO vehicle control within each respective cell line:* indicates *p* < 0.05 in experiments using Z013 cells; # indicates *p* < 0.05 in experiments using iZ013 cells; & indicates *p* < 0.05 in experiments using 3T3-L1 cells. Statistical significance was determined using one-way ANOVA followed by least significant difference multiple comparisons test.

The results of adipogenesis assay showed primary cells (429Z013.1-Male, Z013) and their immortalized derivative (immortalized 429Z013.1-Male, iZ013) had similar responses to the tested chemicals (ROSI and six chemicals) (Fig. 7). ROSI, TBT, TPP, BPA and PFOA consistently increased intracellular lipid accumulation across these two cell types, while p,p’-DDE and TCS showed no effects on either cell type. The results of adipogenesis effects indicated the similar adipogenesis potency between original cells vs. immortalized cells. Overall, both RNA-seq analysis and adipogenesis assay showed strong similarities between the responses of the primary cells and their immortalized derivative, suggesting that these immortalized hBM-MSCs are also suitable for use in this assay.

### 3.9 Comparison of the performance of hBM-MSCs cells and 3T3-L1 cells

The reliability of the hBM-MSCs adipogenesis assay was further assessed by com-paring the results of selected chemicals with those obtained from the widely used 3T3-L1 preadipocyte cell adipogenesis assay. The chemicals tested included ROSI and six selected chemicals. Both hBM-MSCs cell line (429Z013.1-Male) and the 3T3-L1 cell line (3T3-L1) showed significant change of adipogenesis related genes after adipogenesis induction (compare Fig. 6D and 6E). The results of adipogenesis assay showed these two cell lines had similar response to the tested chemicals (ROSI and six chemicals) (Fig. 7). ROSI, TBT, TPP, BPA and PFOA consistently increased intracellular lipid accumulation across these two cell lines. p,p’-DDE and TCS showed no adipogenesis induction effects in hBM-MSCs assay but showed adipogenesis effects in 3T3-L1 cell assay when tested in a much higher concentration. Overall, both gene expression analysis and adipogenesis assay showed the similar results of the hBM-MSCs and 3T3-L1 assay cells, suggesting the reliability of the hBM-MSCs adipogenesis assay.

## 4 Discussion

Here, we developed an adipogenesis assay to evaluate cellular responses to chemical exposure. We have shown that this assay is able to detect effects from known obesogenic chemicals. Factors that impact assay reproducibility, such as cell confluency, the optimization of fluorescence detection methods, and the stability of staining signals are critically important to increase robustness. Furthermore, we have applied the optimized method to screen potential obesogenic chemicals, confirming the assay’s reproducibility across different hBM-MSC batches and comparing them with an immortalized cell line and the well-established 3T3-L1 adipogenesis model.

We investigated several important factors (cell confluency and detection methods) in the hBM-MSCs adipogenesis assay to ensure reliable and reproducible results. We found that varying cell confluency impacted lipid accumulation and the stability of experimental results (Fig. 2). We recommend initiating differentiation with the MDI cocktail and ligands when the cells are just short of complete confluency. We also examined the impact of the number of detection points per well via a bottom read of a well, when assessing Nile Red and Hoechst fluorescence. Based on the results, we recommend detecting at least nine points per well for reliable data (Fig. 3). We evaluated the stability of staining signals over time and found that fluorescence signals could be reliably measured up to 10 days post-staining with proper storage, which provides flexibility in experimental workflows (Fig. 4). By optimizing these experimental conditions, we can better control variables, reduce errors, and obtain more reliable and stable results, which are crucial for the research using the hBM-MSCs adipogenesis assay. In our current study, we focused on the key experimental variables, but we recognize that other factors such as the type of plastic plates and media additives can influence adipogenesis. We suggest that these variables could be explored in future studies to enhance the reproducibility and reliability of the assay.

Cells obtained from different anonymous human donors may exhibit varying adipogenic potential due to biological variability across individuals. This variability may be influenced by factors such as age, gender, genetic background, and metabolic health (e.g., obesity, diabetes). Furthermore, lifestyle factors such as diet and physical activity may also contribute to differences in adipogenic capacity. Thus, it is essential to test this capability when using new hBM-MSCs, irrespective of the source. The protocol employed in this study involved testing the responses of hBM-MSCs to two positive control ligands (ROSI and LG) known to induce adipogenesis. Specific cell lots, such as 429Z001-Female, 438Z012.1-Male, and 429Z013.1-Male, demonstrated adipogenic responses to these two positive control ligands, indicating their suitability for adipogenesis studies (Fig. 4). In contrast, other lots, such as 4272,010.1-Female, 18TL169252-Female, and 4292Z022-Male, showed deficiencies such as lack of response to ROSI and/or LG, or morphological anomalies, making them unsuitable for further experimentation. Our recommendation is to assess the adipogenic potential using positive controls when purchasing new batches of hBM-MSCs.

Based on our results, a new cell method should achieve several criteria to be considered suitable for in vitro adipogenesis assays (Legler et al., 2020). Cell morphology should be like that of the normal hBM-MSCs. The cell should have the ability to differentiate into adipocytes using 500 nM ROSI and 100 nM LG as positive control ligands. According to our results, we suggest that the response to these two positive ligands should be in the range of at least 1.5-fold to 3.5-fold of lipid accumulation compared with DMSO vehicle control. It is important to rigorously test and select hBM-MSC lots based on their adipogenic potential by using positive control ligands (ROSI and LG) before using them to test other chemicals. Such practices are crucial for ensuring the validity and reliability of experimental outcomes.

After optimizing assay conditions, we evaluated the effects of six selected chemicals (Legler et al., 2020) (TBT, TPP, BPA, PFOA, p,p’-DDE, and TCS) on hBM-MSC adipogenesis and investigated the replicability, repeatability and variability of the assay, including different donors (Fig. 3, Fig. 7). Based on the results of ROSI and six selected chemicals by using one cell line, the RSD ranged from 2 % to 27 % for three replicate wells. The RSD ranged from 8 % to 25 % for the effects at highest concentrations tested in three separate experiments. These results suggested good replicability and repeatability of the assay. Our results showed that two of the tested chemicals (TBT and TPP) had the ability to induce adipogenesis, which is consistent with previous studies about their adipogenic induction effects by using MSC, preadipocyte or other cell lines (Tung et al., 2017; Pillai et al., 2014; Pascuali et al., 2024; Ticiani et al., 2023).

BPA and PFOA showed equivocal results between the different batches and replicate runs. In one study, no significant effect on adipogenesis is found at BPA in concentrations ranging from 1 nM to 100 µM after a 14-day exposure period and report cytotoxicity above 100 µM (Chamorro-García et al., 2012). In another study, increased adipogenesis and increased upregulation of adipogenic genes such as PPARγ was found at BPA concentrations of 25 µM and 50 µM, however measured in human preadipocytes (Boucher et al., 2014). Overall, these findings are in line with our results. Even though we did find some significant increases at 100 µM in our study, these results were barely significant, not consistent across replicates and cell batches, and the overall experiment did not pass the 1.5 fold change threshold. Neither p,p’-DDE nor TCS showed any effects in our assay. p,p’-DDE increased lipid accumulation in an earlier study, however, this was with lipid-containing medium in adipose derived stem cells (Pesta et al., 2018), whereas with TCS a slight inhibitory effect on adipogenesis was reported in hMSCs (Guo et al., 2012). Overall, except for the variability observed with PFOA and BPA at very high exposure levels, the results from the three different cell lines were consistent, indicating good assay reliability. PFAS chemicals, especially long chain PFAS, are known to require careful attention due to their amphiphilic nature, which could explain the variability of PFOA. This variability assessment demonstrated the robustness of the assay when applied to primary cells from different donors, ensuring reliable and reproducible outcomes across different sourced hBM-MSCs.

Primary MSCs, while useful for providing a close approximation of in vivo conditions, have several limitations. They exhibit significant donor variability, have a limited lifespan, and often show inconsistent responses across different batches. These limitations can lead to variability in experimental results, making it challenging to obtain reproducible and reliable data. In contrast, immortalized MSC cells offer several advantages. They possess a stable genetic background, extended lifespan, and can produce consistent results across multiple experiments. These characteristics make immortalized MSC cells particularly valuable for high-throughput screening and long-term studies, as they minimize variability and enhance reproducibility. There are several commercially available immortalized human MSC lines (ASC52telo, 2025; Immortalized Human, 2025).

Here, we immortalized one hBM-MSC cell line (429Z013.1-Male) and used it to study the adipogenic induction effects of various chemicals. The results of RNA-seq analysis demonstrated transcriptomal similarity between the primary cells line and their immortalized derivative cell line (Fig. 6A–C). Subsequent testing of the immortalized cell’s adipogenic response to ROSI and six selected chemicals revealed that the immortalized 429Z013 cell line exhibited similar sensitivity compared to the primary cells (Fig. 7). These results showed the similarity between the primary and immortalized cells, suggesting the utility of the immortalized hBM-MSCs used in this assay. Further assessment of the immortalized vs. primary hMSCs cells subject for further studies (Hoffmann et al., unpublished data). The use of immortalized hMSCs represents a potential approach to improve assay consistency and availability. In this study, we observed promising results with one immortalized hMSC line, suggesting possible advantages in terms of reproducibility and scalability. However, as only a single immortalized cell line was evaluated, further validation using additional, commercially available immortalized hMSC lines is necessary. Future studies should systematically assess the performance of such cell lines to determine their suitability for use in adipogenesis assays.

Finally, we compared the performance of the hBM-MSC adipogenesis assay with that of the widely used murine 3T3-L1 adipogenesis assay. Overall, gene expression profiles and responses to ROSI and six selected chemicals in the hBM-MSC assay were largely consistent with those observed in the 3T3-L1 assay (Fig. 6D–Fig. 6, Fig. 7). Our results showed that TBT, TPP, and BPA promoted adipogenic differentiation in hBM-MSCs, consistent with outcomes in 3T3-L1 cells and with prior studies reporting similar effects in both models [25,33,34 for hMSCs; 16,27,35 for 3T3-L1]. For PFOA, our hBM-MSC assay indicated a pro-adipogenic effect, while no such effect was observed in the 3T3-L1 model. Previous reports, however, have shown adipogenesis-promoting activity of PFOA in both hMSCs and 3T3-L1 cells (Watkins et al., 2015; Qin et al., 2022). Conversely, p,p’-DDE did not induce adipogenesis in our hBM-MSC assay but did show an effect in the 3T3-L1 assay—findings that align with literature describing its adipogenic activity in both systems (Mangum et al., 2015; Kladnicka et al., 2021). In the case of TCS, we found no effect in hBM-MSCs, whereas it promoted adipogenesis in 3T3-L1 cells. Prior hMSC studies suggest TCS may inhibit adipogenesis (Guo et al., 2012), while results in 3T3-L1 cells have been inconsistent, with both pro- and anti-adipogenic effects reported (Schmid et al., 2005; Zhang et al., 2024), pointing to model-dependent variability. Although some discrepancies were noted, the general consistency in responses for most compounds supports the reliability of the hBM-MSC adipogenesis assay. Strong adipogenic inducers such as TBT and TPP produced consistent results across all models tested. In contrast, weaker-acting compounds like PFOA and TCS showed more variable effects, which may be attributed to differences in cell type, species origin, or assay sensitivity. These findings underscore the importance of further investigating inter-model variability in chemical responses to better understand their adipogenic potential.

The murine preadipocyte cell line 3T3-L1 is a commonly used model for screening obesogenic substances (Ruiz-Ojeda et al., 2016; Cave and Crowther, 2019; Zebisch et al., 2012). This immortalized cell line offers several benefits, including short experimental duration and suitability for high-throughput screening methods (Poulos et al., 2010). However, a key limitation of 3T3-L1 cells is that they are already committed preadipocytes, making them less suitable for studying mechanisms that involve the commitment of uncommitted cells to the adipocyte lineage. Additionally, as mouse cells, there can be limitations in extrapolating results to human. These findings highlight some limitations of using 3T3-L1 cells as a general model for adipogenesis. Previous studies have demonstrated that both the early commitment of MSCs to adipocytes and their subsequent promotion of adipogenesis can be influenced by obesogens, which makes the uncommitted human MSCs more valuable for comprehensive analyses of how chemicals affect adipogenesis processes compared to the preadipocyte cell lines (Kassotis et al., 2022). Additionally, as primary cells isolated directly from humans, human MSC cultures offer more relevance to human outcomes than some mouse or rat cell lines. Therefore, the hBM-MSCs adipogenesis assay could provide some advantages as a robust method to identify obesogens. Recent studies have also indicated that the commercial source of the cell line, the type of plates used, and the number of cell passages can affect the sensitivity of 3T3-L1 cells to induction of adipogenesis by chemicals (Kassotis et al., 2022). While many variable factors, such as the type of culture plates used and the number of cell passages, were not evaluated in the present study, they may contribute to variability in hMSC-based assays. These factors are well-documented in the literature and should be taken into account in future investigations to enhance the robustness and reproducibility of adipogenesis assays.

## 5 Conclusions

In this study, we established an accurate, reproducible, and sensitive methodology for assessing the adipogenic potential of chemicals using hBM-MSCs. We assessed lipid accumulation, normalized to DNA content, as a reliable endpoint for evaluating adipogenic differentiation. Our findings underscore the importance of ensuring near to, but not 100 % cell confluency, detecting at least nine points per well and demonstrated the stability of fluorescence signals for up to ten days post-staining. Using the established method, we evaluated the effects of six selected chemicals on adipogenesis, the results suggested good replicability and repeatability of the assay. We addressed possible variability among hBM-MSCs from different donors by rigorously testing and selecting suitable cell lots using positive control ligands (ROSI and LG) to ensure consistent experimental outcomes. We recommend doing this assessment each time a new lot of cells is obtained. Recognizing limitations of primary cells, we successfully created an immortalized hBM-MSC cell line. This immortalized line exhibited similar gene expression profiles to primary cells and similar response to adipogenesis-inducing chemicals, enhancing its utility for long-term and high-throughput studies. Comparison with the widely used 3T3-L1 adipogenesis assay showed consistent results across tested chemicals. In conclusion, our optimized hBM-MSC adipogenesis assay provides a reliable and sensitive tool for studying chemical effects on adipogenesis, advancing our understanding of how obesogens contribute to obesity.

##  

Supplementary Materials: Two texts (Prat 1. Detailed standard operating procedure (SOP) of the hBM-MSCs adipogenesis assay and Part 2. Detailed standard operating procedure (SOP) of the 3T3-L1adipogenesis assay), one Figure (Figure S1. Recommended time schedule of the hBM-MSCs adipogenesis assay; Figure S2. Impact of detection point number on the results; Figure S3. Stability of the Nile Red, Hoechst signals and the lipid accumulation detection results.) and two Tables (Table S1. qPCR primer sequences for hBM-MSCs adipogenesis assay and Table S2. qPCR primer sequences for 3T3-L1 adipogenesis assay)

## CRediT authorship contribution statement

**Xiao-Min Ren:** Writing – original draft, Visualization, Validation, Software, Methodology, Investigation, Formal analysis, Data curation. **Richard C Chang:** Investigation, Data curation. **Angélica Amorim Amato:** Investigation, Data curation. **Yikai Huang:** Investigation. **Brittanie Yukimtiao:** Investigation. **Alexandra Esser:** Investigation. **Emma Witteveen:** Investigation. **Juliette Legler:** Supervision, Funding acquisition. **Jorke H Kamstra:** Writing – review & editing, Writing – original draft, Investigation, Data curation. **Bruce Blumberg:** Writing – review & editing, Writing – original draft, Supervision, Resources, Project administration, Funding acquisition, Formal analysis, Conceptualization.

## Declaration of competing interest

The authors declare that they have no known competing financial interests or personal relationships that could have appeared to influence the work reported in this paper.

## Appendix Supplementary materials

Image, application 1
