# Supplementary material for: Development and characterization of a standardized adipogenesis assay for testing metabolism disrupting chemicals using human bone marrow derived mesenchymal stem cells
Source: NAM J. 2025 May 22;1:100029. doi: 10.1016/j.namjnl.2025.100029 (PMC13288643; doi:10.1016/j.namjnl.2025.100029)
Supplement: Supplementary file 1 [file mmc1.docx]

**Supplemental Material**

**Development and Characterization of a Standardized Adipogenesis Assay for Testing Metabolism Disrupting Chemicals Using Human Bone Marrow Derived Mesenchymal Stem Cells**

**Number of pages: 26**

**Two texts：**

**Prat 1.** Detailed standard operating procedure (SOP) of the hBM-MSCs adipogenesis assay

**Part 2.** Detailed standard operating procedure (SOP) of the 3T3-L1adipogenesis assay

**Three Figures:**

**Figure S1.** Recommended time schedule of the hBM-MSCs adipogenesis assay.

**Figure S2.** Impact of detection point number on the results.

**Figure S3.** Stability of the Nile Red, Hoechst signals and the lipid accumulation detection results.

**Two Tables:**

**Table S1.** qPCR primer sequences for hBM-MSCs adipogenesis assay

**Table S2.** qPCR primer sequences for 3T3-L1 adipogenesis assay

**Prat 1. Detailed standard operating procedure (SOP) of the hBM-MSCs adipogenesis assay**

**1.1 Principle of the assay**

Mesenchymal stem cells differentiate into adipocytes through a complex and multi-step process comprising a network of transcription factors that change gene transcription to promote the adipocytic phenotype. These transcription factors include peroxisome proliferator-activated receptor gamma (PPARγ), CCAAT/enhancer-binding proteins (C/EBPs), sterol regulatory element-binding protein (SREBP), and glucocorticoid receptor.

Adipocyte differentiation can be induced in cell culture by stimulation of mesenchymal stem cells with isobutylmethylxanthine (IBMX), dexamethasone, and insulin. IBMX inhibits phosphodiesterases, leading to increased intracellular levels of cyclic AMP (cAMP) and activation of C/EBP-δ, and dexamethasone activates C/EBP-β. C/EBP-β and -δ, in turn, induce the expression of C/EBP-α and PPARγ. Insulin stimulates both adipogenesis and lipogenesis through induction of SREBP-1c and other transcription factors, in addition to directly inhibiting lipolysis. Adipogenesis can be assessed and quantified by determining different endpoints, such as the degree of intracellular lipid accumulation, the expression of genes and their corresponding protein products, and biochemical processes that are characteristic of mature adipocytes.

This standard operating procedure is designed to evaluate the differentiation of human bone marrow-derived mesenchymal stem cells into adipocytes using the adipose induction cocktail consisting of IBMX, dexamethasone, and insulin. The degree of differentiation is assessed by quantifying lipid accumulation by staining neutral lipids with Nile Red, normalized by staining nucleic acid with Hoechst 33342. Data interpretation for induction or inhibition of adipogenesis is based upon lipid accumulation induced by a test chemical compared with control (non-treated cells).

**1.2 Procedure**

***1.2.1 Human Mesenchymal Stem Cells Derived from Bone Marrow***

Pure and sterile primary human mesenchymal stem cells derived from bone marrow (hMSC-BM) should be used for the assay. Only cells with assured multipotency (adipogenesis, chondrogenesis, and osteogenesis) should be used in testing. The cells can be purchased at passage 2 from PromoCell (Germany), in cryopreservation solution.

***1.2.2 Human Mesenchymal Stem Cells Derived from Bone Marrow expansion and freezing***

To make a stock of hMSC-BM, cells can be expanded and then frozen at passage 4. Next, they can be thawed, further expanded, and used at passage 6 for the adipogenesis assay. Upon arrival, cryopreserved hMSC-BM should be immediately stored in liquid nitrogen or seeded at the density of 4,000 cells/cm^2^. To seed cells, MSC growth medium (SupplementMix, MSC Growth Medium 1, PromoCell) supplemented with 100 IU/mL Penicillin, and 100 μg/mL Streptomycin should be used. The following steps can be conducted:

- Step 1. Calculate the needed surface area according to (i) the lot-specific cell number described on the certificate of analysis, and (ii) the plating density of 4,000 cells/cm^2^.
- Step 2. Fill the appropriate cell culture flask with at least 9 mL of MSC growth medium per vial and place it in an incubator for 30 minutes (5% CO_2_, 37^o^C).
- Step 3. Remove the cryovial from the liquid nitrogen container and place it under a laminar flow bench, quarter-turn its cap to relieve pressure and then retighten the cap.
- Step 4. Immerse the cryovial into a water bath (37^o^C) up to below the cap for 2 minutes and then rinse it with 70% ethanol.
- Step 5. In the laminar flow, aspirate the excess ethanol from the area of the cap, open the vial, and transfer the cells to the flask containing prewarmed cell culture medium.
- Step 6. Place the cell culture flask in an incubator (5% CO_2_, 37^o^C) for cell attachment.
- Step 7. Change the MSC growth medium after 3 to 4 hours, and every two to three days thereafter.

The cells should be subcultured or frozen once they have reached 70 to 90% confluency. Subcultivation can be conducted as follows:

- Step 1. Prewarm MSC growth medium and 0.25% trypsin, 2.21 mM EDTA solution in a water bath (37^o^C).
- Step 2. In the laminar flow, carefully aspirate cell culture medium from cell culture flasks.
- Step 3. Add 0.25% trypsin, 2.21 mM EDTA solution (15 μL/cm^2^ of flask surface), close the cell culture flask, and incubate for 2 to 5 minutes at 37^o^C.
- Step 4. Examine the cells under the microscope. When cells start to detach, gently tap the side of the cell culture flask to loosen the remaining cells and take the cell culture flask back to the laminar flow.
- Step 5. Add MSC growth medium to the cell culture flask (9 times the volume of trypsin/EDTA solution used), and carefully resuspend the cells by pipetting up and down.
- Step 6. Count the cells and resuspend them in the appropriate volume of MSC growth medium to seed them at the density of 4,000 cells/cm^2^.
- Step 7. Close the cell culture flasks and place them in an incubator (5% CO_2_, 37^o^C). Change the MSC growth medium after 3 to 4 hours, and every two to three days thereafter.

To freeze the cells, the following steps can be conducted:

- Step 1. Prepare the freezing medium by adding 10% (v/v) DMSO to MSC growth medium.
- Step 2. Prewarm MSC growth medium and 0.25% trypsin, 2.21 mM EDTA solution in a water bath (37^o^C).
- Step 3. In the laminar flow, carefully aspirate cell culture medium from cell culture flasks.
- Step 4. Add 0.25% trypsin, 2.21 mM EDTA solution (15 μL/cm^2^ of flask surface), close the flask, and incubate for 2 to 5 minutes at 37^o^C.
- Step 5. Examine the cells under the microscope. When cells start to detach, gently tap the side of the cell culture flask to loosen the remaining cells, and take the cell culture flask back to the laminar flow.
- Step 6. Add MSC growth medium to the flask (9 times the volume of trypsin/EDTA solution used), and carefully resuspend the cells by pipetting up and down, and count cells.
- Step 7. Spin down the cells for 3 minutes at 220 × *g*.
- Step 8. In the laminar flow, remove the supernatant and resuspend the cells in the appropriate volume of freezing media (1 mL of medium/10^6^ cells) and distribute them in cryovials. Cryovials should be labeled with the origin of cells, lot number, date and passage at which cells were frozen.

***1.2.3 Human Mesenchymal Stem Cells Derived from Bone Marrow seeding for induction of adipogenesis***

Once cells have been initially expanded and frozen at passage 4, they can be thawed, further expanded and used in the adipogenesis assay at passage 6. The medium that can be used to thaw, further expand cells and induce adipogenesis is Minimal Essential Medium α (MEMα), pH 7.3, supplemented with 15% nonstripped fetal bovine serum, 2.2g/L sodium bicarbonate, 20 mM HEPES, 100 IU/mL Penicillin, and 100 μg/mL Streptomycin.

To thaw the cells, the following steps can be conducted:

- Step 1. Prewarm MEMα in a water bath (37^o^C).
- Step 2. Remove the cryovial from the liquid nitrogen container and place it under a laminar flow bench, quarter-turn its cap to relieve pressure and then retighten the cap.
- Step 3. Immerse the cryovial into a water bath (37^o^C) up to below the cap for 2 minutes and then rinse it with 70% ethanol.
- Step 4. In the laminar flow, aspirate the excess ethanol from the area of the cap, open the vial, and transfer the cells to a 15 mL conical tube. Add 10 mL of prewarmed MEMα.
- Step 5. Spin down the cells for 3 minutes at 220 × *g*.
- Step 6. In the laminar flow, remove the supernatant and resuspend cells in the appropriate volume to seed the cells at the density of 4,000 cells/cm^2^.
- Step 7. Place the cell culture flasks in an incubator (5% CO_2_, 37^o^C) for cell attachment.
- Step 8. Change the MEMα every two to three days thereafter.

Upon reaching 70 to 90% confluency, cells can be expanded by subcultivation as previously described, and should be seeded at passage 6 to prepare the assay plates. To seed plates, cells should be collected by using 25% trypsin, 2.21 mM EDTA solution, as described previously, resuspended in prewarmed MEMα (4 × 10^4^ cells/mL), and plated into the wells of 24-well plates, 1 mL of cell suspension/well.

Next, the 24-well plates containing the cells should be incubated in a 5% CO_2_ at 37^o^C until confluency (48-72h), and no more than two plates should be grouped (one on top of each other) in the incubator. After reaching confluency, the cells should be induced with MEMα containing the adipose induction cocktail, in the presence of vehicle control, positive controls (rosiglitazone, LG), and the test chemicals in the desired concentration. MEMα with the assigned exposures is changed every 3 to 4 days for 14 days.

***1.2.4 Preparation of medium with adipose induction cocktail***

To induce differentiation of hMSC-BM into adipocytes, each component of the adipose induction cocktail (IMBX, dexamethasone and human recombinant insulin, MDI) should be prepared and then added to MEMα.

IMBX (0.05 M) stock solution should be prepared fresh, and the following steps should be conducted.

- Step 1. Prepare 0.5 N potassium hydroxide solution and store it at room temperature.
- Step 2. Prepare fresh 0.05 M IMBX solution by weighing between 112 mg to 120 mg of IMBX for each liter of MEMα with MDI to be prepared. The volume (mL) of 0.5 N KOH to make a 0.05 M IMBX solution can be calculated as: volume of KOH = IMBX (mg)/11.112.

Dexamethasone (100 mM) stock solution should be prepared as follows:

- Step 1: prepare a 100 mM dexamethasone in DMSO by weighing approximately 20 mg of dexamethasone and. The volume of DMSO (mL) to make a 100 mM dexamethasone solution can be calculated as: volume of DMSO = dexamethasone (mg)/39.246.
- Step 2. Store 100 mM dexamethasone at -20^o^C, using amber vials. In these conditions, it is stable for 5 years.

Insulin (50 mg/mL) stock solution should be prepared as follows:

- Step 1. Prepare 0.02 N hydrochloric acid solution and store it at room temperature.
- Step 2. Prepare 50 mg/mL insulin solution by weighing approximately 50 mg of insulin. The volume (mL) of 0.02 N HCl to make a 50 mg/mL insulin solution can be calculated as: insulin (mg)/50.
- Step 3. Store 50 mg/mL insulin solution at -20^o^C, using amber vials. In these conditions, it is stable for 5 years.

MEMα with adipose induction cocktail (0.5 mM IBMX, 1 μM dexamethasone, 5 mg/mL insulin) can be prepared as follows:

- Step 1. Add 10 mL of freshly prepared 0.05 M IBMX solution in 0.5 N potassium hydroxide to each liter of MEMα.
- Step 2. Add 10 μL of 100 mM dexamethasone stock solution in DMSO to each liter of MEMα.
- Step 3. Add 1 mL of 5 mg/mL insulin stock solution in 0.02 N hydrochloric acid to each liter of MEMα.
- Step 4. Filter sterilize the medium with adipose induction cocktail through a 0.22 μm filter and store at 4^o^C. The medium can be used for up to one month.

***1.2.5 Vehicle for positive controls and test chemicals***

DMSO is generally used to solubilize the test chemicals and is miscible with the cell culture medium. For any other vehicle that is used, it should be demonstrated that the maximal concentration used is not toxic to hMSC-BM, and that it does not interfere with adipogenesis assay performance, as indicated by response to the positive controls. The concentration of DMSO in MEMα with MDI should not exceed 0.101%, corresponding to 0.001% from dexamethasone stock solution diluted 1,000 times in MEMα, and 0.1% from test chemicals, as will be described in the next topic.

***1.2.6 Preparation of Test Chemicals and Positive Controls***

The test chemicals should be dissolved in DMSO or the appropriate solvent to the soluble concentration corresponding to 1,000 times the highest final concentration (in cell culture medium, MEMα with MDI) desired in the assay. Next, it should be serially diluted to obtain the other desired concentrations to be tested. Maximum concentration should also be selected based on the avoidance of precipitation in cell culture medium.

Rosiglitazone and LG should be used as positive controls for adipogenic induction. Stock solutions for both positive controls are prepared in DMSO at 1,000 times higher concentration than the desired final concentration to expose cells. It is recommended to weigh at least 2 mg to prepare the stock solutions. Stock solutions of rosiglitazone and LG should be stored in amber vials and at -20^o^C.

***1.2.7 Chemical exposure***

DMSO (vehicle control), each positive control, and each test chemical at 1,000 times the desired final concentration in cell culture medium (MEMα with MDI) should be diluted to achieve the final concentration for cell exposure. Exposure to DMSO, each positive control, and each concentration of the test chemicals should be conducted in triplicate, and the recommended final volume of medium for each well is 1 mL.

Preparation of exposures could be conducted as follows:

- Step 1. Label eight 15-mL sterile conical tubes according to the exposures

1. DMSO.
2. 500 nM rosiglitazone.
3. 50 nM TBT.
4. TC conc 1.
5. TC conc 2.
6. TC conc 3.
7. TC conc 4.
8. TC conc 5.

- Step 2. In the laminar flow, add 4 mL of prewarmed MEMα with MDI to each tube. This corresponds to 1 mL of medium for each of the three well receiving the same exposure in the 24-well plate, and 1 mL extra medium.
- Step 3. Add the corresponding exposures to the tubes containing MEMα with MDI:
  1. DMSO tube: 4 μL of DMSO.
  2. 500 nM rosiglitazone: 4 μL of rosiglitazone 50 mM.
  3. 100 nM LG: 4 μL of 10 mM LG.
  4. TC conc 1: 4 μL of TC at 1,000 × concentration 1.
  5. TC conc 2: 4 μL of TC at 1,000 × concentration 2.
  6. TC conc 3: 4 μL of TC at 1,000 × concentration 3.
  7. TC conc 4: 4 μL of TC at 1,000 × concentration 4.
  8. TC conc 5: 4 μL of TC at 1,000 × concentration 5.

For exposure, add 1 mL MEMα with MDI and exposures to each corresponding well, under laminar flow. After adding the exposures, the assay plates should be returned to the 5% CO_2_ incubator at 37^o^C. No more than two plates should be grouped (one on top of each other) in the incubator.

Every three to four days, the propagation cell culture medium with MDI and exposures is aspirated carefully without touching the floor of the well, and fresh MEMα with MDI and exposures prepared as described previously is added. After 14 days, adipogenesis can be assessed by fixing cells and staining intracellular neutral lipids. The assay should be repeated independently at least three times for the same batch of hBM-MSC using vehicle control, positive controls, and the same concentrations of the test chemicals.

***1.2.8 Lipid Accumulation Assessment***

Cells are ready for analysis of intracellular lipid accumulation 14 days after induction of adipocyte differentiation. Lipids are stained with Nile Red, and nucleic is stained with Hoechst 33342 to normalize lipid staining for cell count.

Before staining, cells should be fixed in 3.7% formaldehyde, and fixation can be conducted as follows:

- Step 1: Prepare 1×PBS and 3.7% formaldehyde in 1×PBS (500 μL of 3.7% formaldehyde/well).
- Step 2: Carefully aspirate cell culture media without touching the floor of wells.
- Step 3: Wash cells once with 1×PBS.
- Step 4: Fix the cells (500 μL of 3.7% formaldehyde/well) for 30 min at room temperature.
- Step 5: Remove 3.7% formaldehyde by careful aspiration and wash cells twice with 1×PBS. Leave cells in 1×PBS (500 μL/well) at 4^o^C overnight, if phenol red-containing media was used. This is important because phenol red and Nile Red have the same excitation and emission spectra, and by keeping the cells overnight in PBS phenol red is released and does not interfere with Nile Red detection.

After fixation, fluorescence background should be measured. Excitation and emission wavelengths of Hoechst 33342 are 355 nm and 460 nm, respectively. Excitation and emission wavelengths of Nile Red are 485 nm and 590 nm, respectively. If phenol red-containing media was used, remove 1×PBS in which cells were left, by careful aspiration, and replace it with fresh 1×PBS.

After fluorescence background measurement, cells should be stained with Hoechst 33342 and Nile Red, and staining can be conducted as follows:

- Step 1: Prepare stock solutions of Nile Red (1 mg/mL, in DMSO) and Hoechst 33342 (5 mg/mL in MilliQ water). Nile Red stock solution should be kept at -20^o^C and prepared monthly, and Hoechst 33342 solution should be kept at -20^o^C and is stable for several months.
- Step 2: Prepare a work solution containing both 1 μg/mL Nile Red and 5 μg/mL Hoechst 33342 by diluting the stock solutions of Nile Red and Hoechst 1,000 times in 1×PBS. The volume of the work solution is calculated considering that 500 μL/well are used.
- Step 3: Remove 1×PBS from the wells and add 500 μL of the work solution containing Nile Red and Hoechst to each well, and stain cells for 30 minutes at room temperature.
- Step 4: Remove the work solution of Nile Red and Hoechst and wash cells twice with 1×PBS.
- Step 5: Measure Nile Red and Hoechst fluorescence using the same excitation and emission wavelengths.

Preparation of stock and work solutions of Nile Red and Hoechst should be conducted in the dark, and the solutions should be kept in amber vials. Staining should also be conducted in the dark and the assay plates should be protected from light until analysis. This can be accomplished by wrapping plates in aluminum foil.

***1.2.9 Data Analysis***

Intracellular lipid accumulation is calculated by normalizing Nile Red relative fluorescence units to Hoechst 33342 relative fluorescence units, according to the following steps:

- Step 1. Calculate the average background fluorescence and Nile Red and Hoechst fluorescence for each well.
- Step 2. Subtract the average background fluorescence from the corresponding Nile Red and Hoechst fluorescence for each well.
- Step 3: Divide Nile Red fluorescence for Hoechst fluorescence.

***1.2.10 Material, Reagents and Chemicals used in Adipogenesis Assay***

| **Chemical/reagent** | **Company** | **Catalog number** |
| --- | --- | --- |
| 24-well plate, Nunclon Delta Surface | ThermoScientific | 142475 |
| 75-cm^2^ flask | Corning | 353136 |
| Cryo 1^o^C Freezing Container | Nalgene | 5100-0001 |
| Dexamethasone | Sigma-Aldrich | D4902 |
| DMSO | Fisher BioReagents | BP231-100 |
| Fetal bovine serum | e.g., Gemini Bio-Products – qualified as in Appendix III | 100-500 |
| Formaldehyde | Fisher Chemical | F79-1 |
| HEPES | Fisher Chemical | BP310-1 |
| Hoechst 33342 | Invitrogen | H21492 |
| Insulin | Sigma-Aldrich | 91077C |
| Isobutylmethylxanthine | Sigma-Aldrich | I5879 |
| Minimal Essential Medium α | Gibco, ThermoFisher Scientific | 12000-022 |
| Nile Red | Sigma-Aldrich | N3013 |
| Penicillin 5,000 IU/mL-Streptomycin 5,000 μg/mL | Corning | 30-001-cl |
| Rosiglitazone | Cayman Chemical Company | 71740 |
| Sodium bicarbonate | Fisher Chemical | S233-500 |
| Mesenchymal Stem Cell Growth Medium 2 | PromoCell | C-28009 |
| Tributyltin chloride | Sigma-Aldrich | T50202 |
| Trypsin solution | Corning | 25-053-cl |
| LGD100268 | Sigma-Aldrich | SML0279 |
| Perfluorooctanoic acid | Sigma-Aldrich | 171468 |
| Triphenyl phosphate | Sigma-Aldrich | 241288 |
| p,p’-Dichlorodiphenyldichloroethylene | Sigma-Aldrich | 43537 |
| Triclosan | Sigma-Aldrich | 72779 |
| Bisphnol A | Sigma-Aldrich | 239658 |

**Part 2. Detailed standard operating procedure (SOP) of the 3T3-L1adipogenesis assay**

**2.1 Principle of the assay**

3T3-L1 preadipocytes differentiate into adipocytes through several waves of transcription factor activation. Once CAAT/enhancer-binding proteins β (C/EBPβ) and C/EBPδ are activated by an adipogenic cocktail the cells expand for 1 cell cycle followed by growth arrest. A second wave of transcription factor activation involves, including C/EBPα and peroxisome proliferator activated receptor γ (PPARγ) (which forms a heterodimer with retinoic X receptor α (RXRα)), induced gene expression of many late acting genes involved in lipid biosynthesis and glucose transport (e.g. fatty acid binding protein 4 (FABP4), glucose transporter type 4 (SLC2A4), and adiponectin (ADIPOQ).

The adipogenic cocktail consists of isobutylmethylxanthine (IBMX), dexamethasone, and insulin. Dexamethasone activates C/EBP-β and IBMX inhibits phosphodiesterases. In 3T3-L1, the use of dexamethasone is omitted to reduce background differentiation. Insulin stimulates both adipogenesis and lipogenesis through induction of SREBP-1c and other transcription factors, in addition to directly inhibiting lipolysis. Adipogenesis can be assessed and quantified by determining different endpoints, such as the degree of intracellular lipid accumulation, the expression of genes and their corresponding protein products, and biochemical processes that are characteristic of mature adipocytes.

This standard operating procedure is designed to evaluate the differentiation of 3T3-L1 cells into adipocytes. The degree of differentiation is assessed by quantifying lipid accumulation by staining neutral lipids with Nile Red, normalized by staining nucleic acid with DAPI. Nile Red also stains phospholipids in the cell membrane. As a consequence Nile Red is not completely specific for neutral lipids and does have some fluorescent activity in all cell types. Using an excitation wavelength of 485 and an excitation wavelength of 572, reduces the influences of phospholipids. Data interpretation for induction or inhibition of adipogenesis is based upon lipid accumulation induced by a test chemical compared with solvent control (non-treated cells).

**2.2 Procedure**

## *2.2.1 Cell culture*

### *2.2.1.1 Reagents*

- Medium:

500 mL DMEM with high glucose, Gibco, Cat. No: 11995)

+ 5.55 mL of Penicillin-Streptomycin (10,000 U/mL), Gibco, Cat. No: 15140122

+ 50 mL BCS (bovine calf serum, ATCC: Cat. No: 30-2030)

+ 5.5 mL 100×Glutamax (Gibco, Cat. No: 35050-061)

### *2.2.1.2 Thawing of cells*

1. Warm up medium to 37°C
2. Thaw cells quickly (≈500k cells/ml) in a water bath at 37°C
3. Transfer cells to 10 mL of warm culture medium in 15 mL tube
4. Centrifuge 300 g, 5 min, RT
5. Discard supernatant and resuspend cells in 10 mL medium and transfer to a T75 culture flask
6. Change medium on the next day
7. Grow to 70% confluence

### *2.2.1.3 Sub-culturing cells*

1. Place Medium, PBS and 0.05% Trypsin-EDTA into the water bath to warm for ~20-30 minutes.
2. Remove a flask of 3T3-L1 cells from the incubator and place under microscope to determine confluency.
3. Place flask in hood, and aspirate all medium.
4. Add 10 mL warm PBS to the flask, on side opposite cell growth. Gently rock the flask to rinse the cells.
5. After removing the PBS, add 2 mL 0.05% Trypsin-EDTA to the flask. Rock over cells, and remove 1.5 mL. Incubate the remaining 0.5 mL volume at 37 °C and 5% CO_2_ for 5 minutes.
6. After 5 minutes, check the cells for detachment. Incubate longer if necessary.
7. Gently tap the culture flask to completely detach the cells. Add 10 mL of medium. Gently pipette the mixture up and down to ensure homogeneity.
8. Obtain a sterile T75 flask and label with the following:
9. Cell type
10. Passage number
11. Date
12. Used batch (only necessary if using multiple batches)
13. Initials
14. Pipette the cell mixture into a fresh T75 flask following the table 1 in a final volume of 10 mL.
15. Place flask in incubator set at 37 °C and 5% CO2 .

Important notes:

- Never allow culture to become completely confluent. Subculture BEFORE cultures become 70-80% confluent in order to allow proper differentiation
- 3T3-L1 cells lose differentiation sensitivity over time. As such, they should not be used past passage 10 (this depends on the batch and supplier and needs to be assessed per laboratory). Freeze stocks early and often, and thaw new ones as P10 is reached.

## *2.2.2 Differentiation protocol*

Cells are differentiated using an 8 day differentiation protocol as described below. Note that in some experiments dexamethasone is used in differentiation medium. In the experiment section the differentiation medium used in that experiment can be found. The 8 day differentiation protocol starts 4 days after plating the cells on a wells plate when the cells have grown confluent.

### *2.2.2.1 Reagents*

- Phenol Red Free Medium:

500 mL DMEM with high glucose, without phenol red, Gibco, Cat. No: 31053-028)

+ 5.55 mL of Penicillin-Streptomycin (10,000 U/mL), Gibco, Cat. No: 15140122

+ 50 mL FBS (foetal bovine serum, Gibco: Cat. No: 10270106)

+ 5.5 mL 100×Glutamax (Gibco, Cat. No: 35050-061)

- 0.5M IBMX (Sigma, Cat. No: I5879) stock in 1 M KOH (freshly made)

IMBX (0.5 M) should be prepared fresh, and the following steps should be conducted:

- Prepare 1 M potassium hydroxide solution (Sigma, Cat. No: ) and store it at room temperature.
- Calculate how much is needed for every experiment (one 48 well plate requires 24 mL of induction medium, so 60 mL in total for 2 days, see below). 0.5M IBMX needs to be diluted 1000×, so for every 50 mL, 50 µL is needed

🡪 Always make extra for pipetting issues

- 1. The **weight** for IBMX to make a 0.5 M IBMX solution can be calculated as:

IBMX (mg) = Volume of media needed (L)*111

- 1. The **volume** (mL) of 1M KOH to make a 0.5 M IMBX solution can be calculated as:

V of KOH solution (mL) = IMBX (mg)/111.

- Insulin (Human recombinant, Sigma, I9278, appr. 10 mg/mL)
- Dilute insulin to 1 μg/mL in IM or MM (see below)
- Induction medium (IM)
- Phenol Red Free Medium

+ 0.5 mM IBMX (add from the 0.5 M stock in 1 M KOH, dilute 1000×)

+ 1 µg/mL insulin

- Maintenance medium (MM)
- Phenol Red Free Medium

+ 1 µg/mL insulin

### *2.2.2.2 Seeding of cells (Thursday or Friday (differentiation day -4 or -3))*

1. Place Phenol Red Free Medium, Culture medium, PBS and 0.05% Trypsin-EDTA into the water bath to warm for ~20-30 minutes.
2. Remove a flask of 3T3-L1 cells from the incubator and place under microscope to determine confluency.
3. Place flask in hood, and aspirate all medium.
4. Add 10 mL warm PBS to the flask, on the side opposite of the cell layer. Gently rock the flask to rinse the cells.
5. After removing the PBS, add 2 mL 0.05% Trypsin-EDTA to the flask. Rock over cells, and remove 1.5 mL. Incubate the remaining 0.5 mL volume at 37 °C and 5% CO_2_ for 5 minutes.
6. After 5 minutes, check the cells for detachment. Incubate longer if necessary.
7. Gently tap the culture flask to completely detach the cells. Add 10 mL of Culture Medium. Gently pipette the mixture up and down to ensure homogeneity.
8. OPTIONAL: Subculture a part of the cell suspension in Culture Medium.
9. Transfer the remaining cell suspension in a 15 mL tube and centrifuge for 5 min at 300g at room temperature.
10. Aspirate the medium and resuspend the cell pellet in 10 mL Phenol Red Free Medium in a 50 mL tube
11. Add 10 μL of the cell suspension to each side of a haemocytometer and use microscope to count the cells.
12. Count the four full squares on the top and the bottom, for a total of 8.
13. Record all eight values and average them.
14. Spray slide and haemocytometer with ethanol and clean it.
15. Calculate the average number of cells/square, then multiply by 1E4 (1×10^4) for amounts of cells/mL.
16. Dilute the cells to 7200 cells/mL (Thursday) or 14400 cells/mL (Friday).
17. Seed the amount of 48 well plates needed with 500 μL of diluted cell suspension.
18. Incubate the plates for 3 or 4 days, until 100 % confluency is reached.

### *2.2.2.3 Induction of Differentiation (Day 0, Monday)*

1. Prepare fresh IM by calculating the amount needed. The IM can be used during the experiment (1 refreshment after 24 hrs).
2. Make exposure IM of the chemicals following appendix 1 in 1.8 mL IM.
3. Expose the 48 well plate by aspirating the medium and add 500 μL of exposure IM (always use at least three replicates per chemical)
4. Store the remaining exposure IM at 4 ⁰C in the dark.
5. Incubate the plate for 24 hours and refresh with the remaining exposure IM.
6. Incubate for another 24 hours.

### *2.2.2.4 Maintenance of cells (Day 2, Wednesday)*

1. Prepare fresh MM by calculating the amount needed. The MM can be used during the experiment (1 refreshment at day 4 and day 7).
2. Make exposure MM of the chemicals in 3 mL MM.
3. Expose the 48 well plate by aspirating the medium and add 500 μL of exposure MM (always use at least three replicates per chemical.
4. Store the remaining exposure MM at 4 ⁰C in the dark.
5. Incubate the plate for 2 days and refresh with the remaining exposure MM.
6. Incubate the plate for another 3 days and refresh with exposure MM.

### *2.2.2.5 Staining the cells (Day 8, Tuesday)*

### Reagents:

- 1×PBS -Mg and -Ca (Gibco, 14190)
- 4% Paraformaldehyde (PFA)

Dilute 1 ampule paraformaldehyde 8% (Catalogue no 157-8, Electron Microscopy Sciences) 1:1 with PBS (Gibco 14190 - CaCl2  - MgCl2​ )

- DAPI stock (5 mg/mL, Thermo Fischer, D1306)

1. Dissolve the DAPI (10 mg) in 2 mL of milli Q water
2. Aliquot in portions of 20 μL and store at -20 ⁰C

- DAPI working solution (100 μg/mL, stable at 4 ⁰C for 1 month)

1. Dissolve a 20 μL aliquot of 5 mg/mL stock in 1 mL milli Q water

- Nile Red stock (1 mg/mL in DMSO, Sigma, 72485)

1. Weight approximately 5 mg of Nile Red in a amber glass vial
2. Dissolve in DMSO at a final concentration of 1 mg/mL
3. Store at RT in the dark (stable for 5 years)

- Staining solution (1 µg/mL Nile Red and 1 µg/mL DAPI)

### Fixation and staining

1. After exposure add directly 500 µL PFA to each well by carefully pipetting above the well dropwise.
2. Incubate at RT for 5 minutes.
3. Discard the medium/fixative by emptying the plate upside down over a waste container.
4. Add carefully another 500 µL fixative to each well.
5. Incubate another 10 min.
6. Discard the fixative.
7. Rinse the cells with 200 µL of 1×PBS and discard the PBS by emptying the plate upside down over a waste container.
8. Rinse the cells with 200 µL of milli Q water and discard by emptying the plate upside down over a waste container.
9. Add 200 µL of milli Q water
10. For the background, measure directly on a fluorimeter at the wave lengths shown in table 1 using a bottom scan with at least 40 measurements per well.
11. Discard the milli Q water by emptying the plate upside down over a waste container.
12. Add 200 µL of staining solution to each well.
13. Incubate for 20 minutes in the dark.
14. Discard the staining solution by emptying the plate upside down over a waste container.
15. Rinse the cells twice with 200 µL of milli Q water and discard the milli Q water by emptying the plate upside down over a waste container.
16. Add 200 µL of milli Q water.
17. Cover the plate with aluminium foil and store at 4ºC or measure directly on a fluorimeter using a bottom scan with at least 40 measurements per well. Excitation and emission wavelengths of DAPI are 355 nm and 460 nm, respectively. Excitation and emission wavelengths of Nile Red are 485 nm and 590 nm, respectively.

## *2.2.3 Data Analysis*

Intracellular lipid accumulation is calculated by normalizing Nile Red relative fluorescence units to DAPI relative fluorescence units, according to the following steps:

1. Calculate the average background fluorescence and Nile Red and DAPI fluorescence for each well.
2. The average background fluorescence from the corresponding Nile Red and DAPI fluorescence for each well.
3. Divide Nile Red fluorescence for DAPI fluorescence.


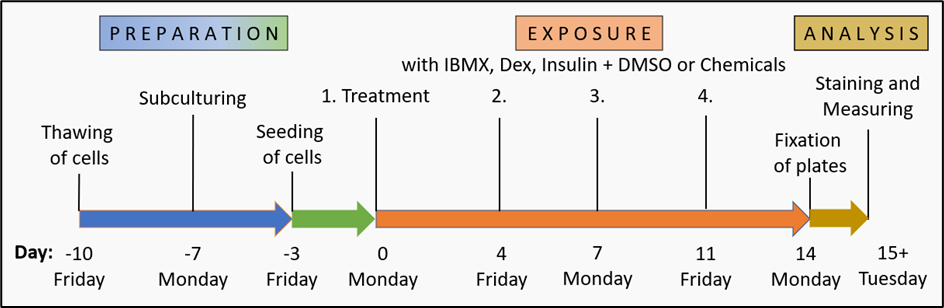


**Figure S1.** Recommended time schedule of the hBM-MSCs adipogenesis assay.

**Figure S2.** Impact of detection point number on the results. The result showed the fold of lipid accumulation of 500 nM ROSI and 100 nM LG compared to DMSO group when detected by the fluorescence plate reader with different detection number per well. * means *p*<0.05, which shows the significant difference of the fold of lipid accumulation of 500 nM ROSI and 100 nM LG compared to DMSO control at the corresponding detection point number.

**
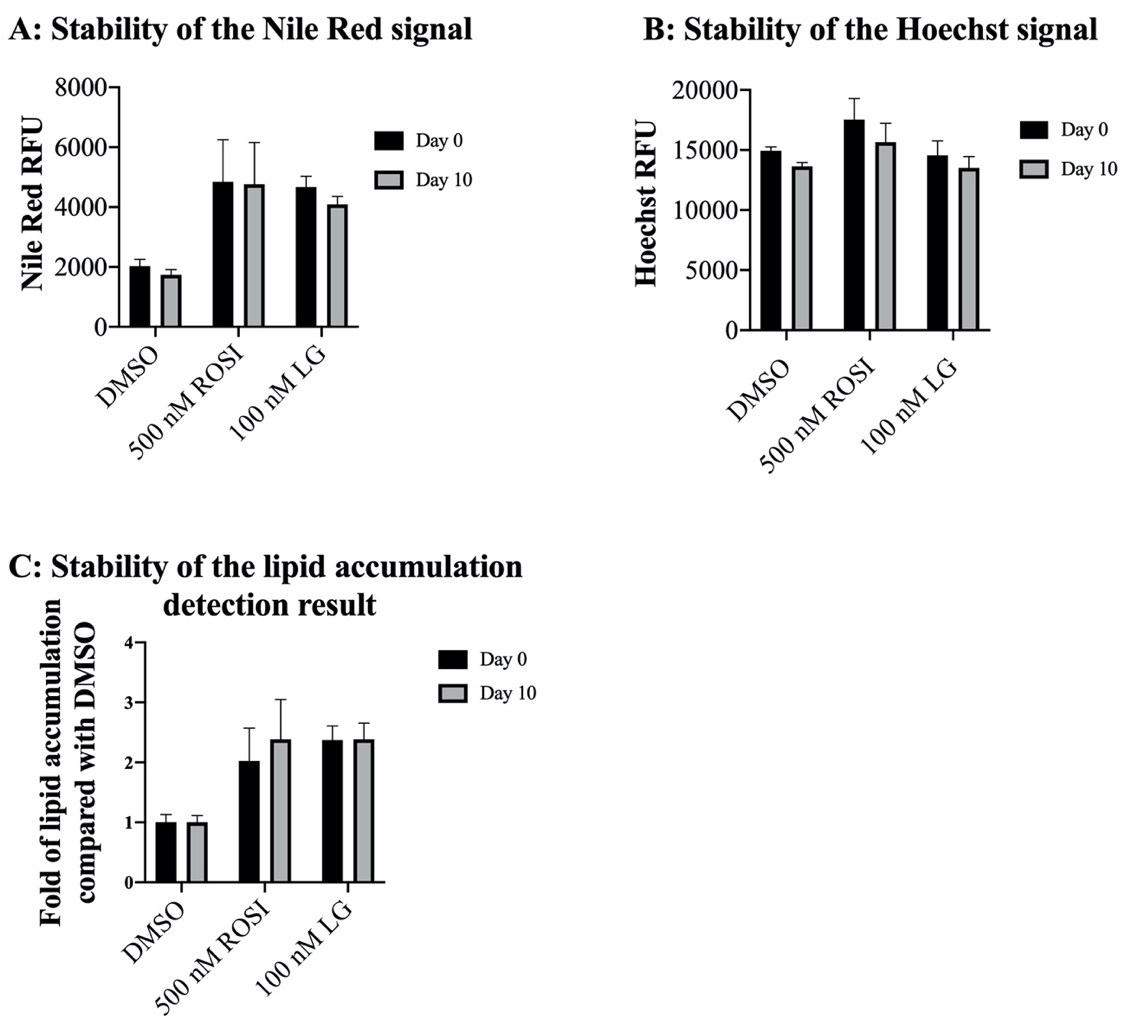
**

**Figure S3.** Stability of the Nile Red, Hoechst signals and the lipid accumulation detection results. A: The signals of Nile Red detected immediately after staining or being stored at 4°C for 10 days; B: The signals of Hoechst detected immediately after staining or being stored at 4°C for 10 days; C: The results of lipid accumulation detection result obtained immediately after staining or being stored at 4°C for 10 days.

**Table S1.** qPCR primer sequences for hBM-MSCs adipogenesis assay

| **Target name** | **Forward Sequence** | **Reverse Sequence** |
| --- | --- | --- |
| FABP4 | AAACTGGTGGTGGAATGCGT | GCGAACTTCAGTCCAGGTCA |
| CEBPa | TATAGGCTGGGCTTCCCCTT | AGCTTTCTGGTGTGACTCGG |
| FSP27 | GCTGTGAGGGGGAGAATGTT | CCCATGGGTCCTTGAGCAAT |
| PPARγ | GCGATTCCTTCACTGATAC | TCAAAGGAGTGGGAGTGGTC |
| ADIPOQ | TCCATACCAGAGGGGCTCAG | GAGTCGTGGTTTCCTGGTCA |
| SLC2A4 | CGACCAGCATCTTCGAGACA | CACCAACAACACCGAGACCA |
| RXRa | TTTCCTGCCGCTCGATTTCT | AGCTGATGACCGAGAAAGGC |
| LPL | CAGGATGTGGCCCGGTTTAT | GCTGGATCGAGGCCAGTAAT |
| INSR | GGCGATATGGTGATGAGGAGC | CTGTCACGTAGAAATAGGTGGGT |
| IGFR | ACGAGTGGAGAAATCTGCGG | ATGTGGAGGTAGCCCTCGAT |
| BACTIN | GAGCACAGAGCCTCGCC | TCATCATCCATGGTGAGCTGG |
| NONO | TCGGTAGAGGAGAAGTCGAGG | CTCTGCATTTTTGCACCCTCA |

**Table S2.** qPCR primer sequences for 3T3-L1 adipogenesis assay

| **Target name** | **Forward Sequence** | **Reverse Sequence** |
| --- | --- | --- |
| Bactin | GTTGGTTGGAGCAAACAT | CATGGATACTTGGAATGACTATT |
| Nono | CATCTAGCTGAAGCTGCC | GATCCAGACAGACCTCCTT |
| RXRa | CACAGACAGACACTTCCT | AGTCAGTTCACAGTCAGAG |
| Fabp4 | GGTGGTGGAATGTGTTATG | ATTGCTTGCTTATTAGTGGAA |
| Slc2a4 | CCCAGTACAGAACTTGAATAC | CTAAAGTGCTGTAGAGGAAAG |
| Lep | GACTCCACAATGCTTGAC | TATCTCTACTTGCTGAAGAACTA |
| Pparγ2 | CTGTTATGGGTGAAACTCTG | ATGGCATCTCTGTGTCAA |
| Ir | TTGTCTCAAAGGGCTGAA | ACTCTGATTGTGCTTCTGA |
| Igfr | AGATCCTGTGTTCTTCTATGT | ACCTGCTGTTATTTCTCTTTC |
| Lpl | CACACTTCTAACACATCACAT | GCAGTTTACAAGCATCCATA |
